# Outstanding Humidity Chemiresistors Based on Imine-Linked Covalent Organic Framework Films for Human Respiration Monitoring

**DOI:** 10.1007/s40820-023-01107-4

**Published:** 2023-06-07

**Authors:** Xiyu Chen, Lingwei Kong, Jaafar Abdul-Aziz Mehrez, Chao Fan, Wenjing Quan, Yongwei Zhang, Min Zeng, Jianhua Yang, Nantao Hu, Yanjie Su, Hao Wei, Zhi Yang

**Affiliations:** Key Laboratory of Thin Film and Microfabrication (Ministry of Education), Department of Micro/Nano Electronics, School of Electronic Information and Electrical Engineering, Shanghai Jiao Tong University, Shanghai, 200240 People’s Republic of China

**Keywords:** Covalent organic frameworks, Humidity sensors, Reversible tautomerism, Non-invasive diagnosis, Health monitoring

## Abstract

Human metabolite moisture detection is important in health monitoring and non-invasive diagnosis. However, ultra-sensitive quantitative extraction of respiration information in real-time remains a great challenge. Herein, chemiresistors based on imine-linked covalent organic framework (COF) films with dual-active sites are fabricated to address this issue, which demonstrates an amplified humidity-sensing signal performance. By regulation of monomers and functional groups, these COF films can be pre-engineered to achieve high response, wide detection range, fast response, and recovery time. Under the condition of relative humidity ranging from 13 to 98%, the COF_TAPB-DHTA_ film-based humidity sensor exhibits outstanding humidity sensing performance with an expanded response value of 390 times. Furthermore, the response values of the COF film-based sensor are highly linear to the relative humidity in the range below 60%, reflecting a quantitative sensing mechanism at the molecular level. Based on the dual-site adsorption of the (–C=N–) and (C–N) stretching vibrations, the reversible tautomerism induced by hydrogen bonding with water molecules is demonstrated to be the main intrinsic mechanism for this effective humidity detection. In addition, the synthesized COF films can be further exploited to effectively detect human nasal and oral breathing as well as fabric permeability, which will inspire novel designs for effective humidity-detection devices.

**Supplementary Information:**

The online version contains supplementary material available at 10.1007/s40820-023-01107-4.

## Highlights

- Imine groups in covalent organic framework (COF) films act as dual-active sites for humidity sensing, inducing an intrinsic enhanced mechanism of reversible protonated tautomerism via water molecule-induced hydrogen bonding.
- The* cis*-ketoimine reciprocal isomerization induces a stretching vibration effect for the ordered conjugated conductive frame of COF films, realizing fast response, wide range, and high sensitivity characteristics for humidity detection.
- Resistance changes of COF film-based sensors keep a strong linear relationship with low-range relative humidity, reflecting the quantitative sensing mechanism at the molecular level.

**Supplementary Information:**

The online version contains supplementary material available at 10.1007/s40820-023-01107-4.

## Graphical abstract

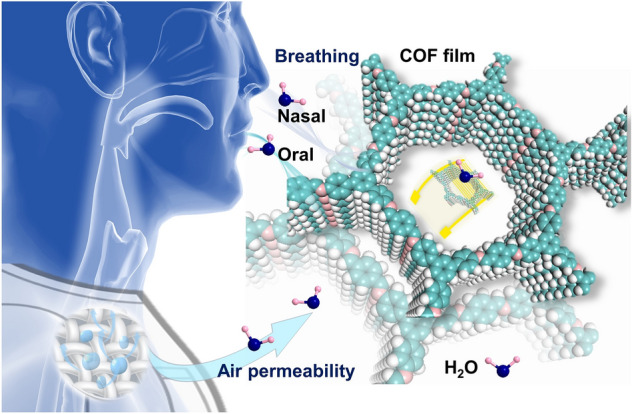

## Introduction

Human metabolism is rich in information and involves water molecules, ranging from skin moisturization to more complex internal respiration that can shed light on the internal health of the human body. Particularly, data from the respiratory tract can represent the physiological and psychological state of the individual, and even the early diagnosis of some diseases (such as lung cancer, epidemic diseases, and apnea syndrome) [1, 2]. Recent studies have demonstrated that respiratory viruses may enhance aerosol transmission risks and are susceptible to large outbreaks, even in lower-humidity environments [3]. Additionally, doctors can use perspiration to diagnose some diseases, uncover drugs, and analyze the performance of athletes [4, 5]. Eventually, to satisfy the quest for practical application, developing a humidity sensor with ultra-sensitivity, real-time quantitative analysis, and a wide detection range is necessary.

Two-dimensional (2D) materials with distinctive microstructures and unique electrical characteristics have attracted great interest in the field of humidity sensing. For example, nanocarbon materials [6, 7], conducting polymers [8], metal–organic frameworks [9], transition metal dichalcogenides [10], and transition metal carbides/nitrides/carbonitrides [11, 12] were investigated as sensing materials. In general, the microstructure of these humidity-sensitive materials can not be precisely designed at the molecular level to obtain optimized sensitivity. To address this issue, a class of porous covalent organic frameworks (COFs) is explored with distinct advantages in the sensing field [13], profiting from the structural stability in complex environments and abundant active sites [14]. The pre-designed COFs can form single or hetero-porous frameworks by selecting monomer linkers and functional groups according to sensing requirements [15].

The chemical affinity between COFs and target analyte is generated by electron-rich/deficient building units to a periodic lattice of donor–acceptor pairs that lead to the stimulated response. Replicating multiple identical binding sites through the whole extended topology shows that the signal can be effectively transduced and amplified through the COFs to achieve a high sensitivity [16]. The benefits of COFs for sensing applications include not only molecule identification but also charge transfer rate acceleration. Furthermore, COFs manufactured into films with continuous pores can expose the greater interior surface area to the analytes, resulting in enhanced sensing performance [17]. It is noteworthy that the strong non-covalent interactions between COF materials and target molecules make them highly selective [18], allowing for reversible colorimetric sensing *via* the inherent linking chemistries as the sensing site, employing complex electron transfer and reversible protonation of the linkage groups [19]. However, it is critical to develop COF-based humidity chemiresistors, especially COF films with (1) higher sensitivity and easier quantification ability; (2) better conductivity, and (3) electronic equipment compatibility for the increasing needs of wearable electronics and health monitoring [20]. For example, the BTA-TAPT COF film-based interdigitated electrodes (IDEs) capacitive sensor exhibits high sensitivity toward benzene vapor [21]. Similarly, since water and benzene molecules can have host–guest interactions with COFs, the researchers developed COF-based electrical humidity sensors. The response and recovery times of the COF-TXDBA-based %RH sensor were 37 and 42 s, respectively [22]. Nevertheless, the response/recovery time of dozens of seconds reported in previous studies remains insufficient for overcoming the limitation of the COFs-based sensor response to human metabolism-related humidity detection.

Herein, we present imine-linked COF films for ultra-sensitive chemiresistive humidity sensors with a wide detection range. We choose the 1,3,5-tris(4-aminophenyl)benzene (TAPB) as the electron-rich structure in order to induce charge exchange between water molecules. On the other hand, the existence of 2,5-dihydroxyterephthaldehyde (DHTA) compared to other monomers such as 4,4′-biphenyldicarboxaldehyde (BPDA) and 1,4-phthalaldehyde (PDA), with strong intramolecular O–H···N=C hydrogen bonding can protect the imine bond from any nucleophilic attacks or the construction of an intact crystalline film. The pre-designed COF films can powerfully facilitate the diffusion of water molecules into and out of the porous framework, thus increasing the response to changes in human breath [23, 24]. Besides, the flexible and free-standing COF films can be easily integrated into humidity detection devices with higher stability. The results indicate that the current amplitude of the humidity sensor based on COF film has a 390-fold increase in a humidity range of 13–98% and a fast response/recovery time of 0.4/1.0 s. Notably, the imine groups of COF films acted as dual-site, and the intrinsic sensing mechanism of hydrogen bonding caused reversible protonated tautomerism for this excellent humidity sensing performance. The humidity sensing mechanism is confirmed by in situ Raman spectroscopy and density functional theory (DFT) calculations. Furthermore, we apply COF film-based sensors to monitor human breathing and the breathability of certain fabrics. The precise molecular structure regulation and continuous porous properties of COF films can accurately capture moisture perturbations. Therefore, the COF films developed in this work hold great promise for ultra-sensitive human-related humidity detection.

## Experimental and Calculation

### Materials and Apparatus

Materials, COF synthesis route and apparatus involved in this work are all represented in the Supporting Information.

### COF Films Design

Electron-rich structures, such as TAPB, were selected as the basis for humidity-induced electrical signals via electron exchange with water molecules. Designed active sensing sites are represented by the N atoms of DHTA which is responsible for the inducement of tautomerism [25]. Water molecules combined with electron-withdrawing groups are supposed to produce group dissociation leading to the formation of a charge transfer complex across the conjugated imine bonds. Moreover, the existence of DHTA with strong intramolecular O–H···N=C hydrogen bonding can protect the imine bond from any nucleophilic attacks or the construction of an intact crystalline film [26]. Given the above considerations, we chose three increasingly electron-rich TAPB, namely 1,3,5-tris-(4-aminophenyl)triazine (TAPT) and 1,3,6,8-tetrakis(4-aminophenyl)pyrene (TAPPy) to provide the polarized electron-accepting imine-linker blocks. These three films referred to as COF_X-DHTA_ are shown in Fig. S1, with their corresponding typologies and synthesis methods.

### Growth Process of COF Films

**Fig. 1:**
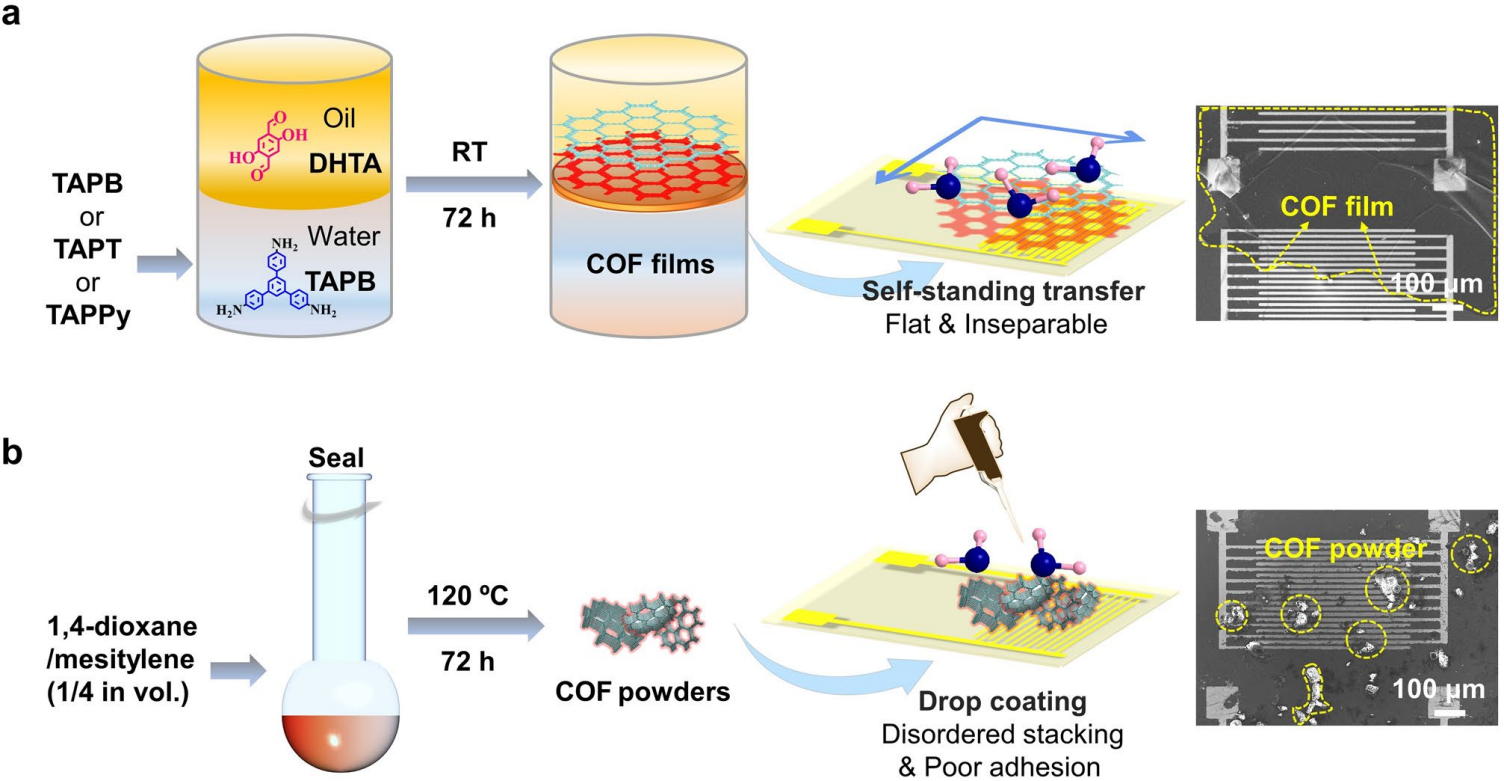
Schematic illustration of the preparation process of the humidity sensor based on the COF **a** films (tightly adhered) and **b** powders (randomly dispersed)

The growth of COF into continuous porous films allows large-scale preparation, and the films are more convenient for processing electronic devices. Inspired by the COFs separation membrane preparation [27], the connecting monomers undergo an imine condensation process at the two-phase interface, resulting in the self-assembly of functional COF films at room temperature (RT). Figure 1 depicts the COF powder- and film-based sensor methods to illustrate the characteristics of the two types of COF sensors. Following a washing procedure, the COF films were directly transferred to the hydrophilic-treated IDEs and assembled into a chemiresistive sensor for subsequent sensing applications. It is critical to ensure that oxygen is isolated throughout the whole process to obtain highly crystalline COF films. The similar-structured monomers TAPT or TAPPy were also successfully synthesized into COF films, demonstrating the generality of the large-scale preparation method. Figure S2 shows the optical photos of COF films prepared by interfacial synthesis and self-assembly. Due to the disorder-to-order transition of the grain boundary, the as-prepared COF_X-DHTA_ films have significantly higher carrier transport and mobilities than powders, resulting in a remarkable humidity-sensing ability. Simultaneously, to speculate the hypothesis that DHTA is the active functional group in humidity sensing, comparative COF films containing BPDA and PDA were prepared (Figs. S3 and S4). Moreover, to determine whether the thickness of the COF film affects sensing capability, a series of COF_TAPB-DHTA_ films of varying thicknesses were prepared by regulating the condensation reaction of a constant amount of TAPB with varied masses of DHTA monomer.

### Humidity-Sensing Measurements

The advantages of COF films will be demonstrated in the following humidity performance tests. The preparation of the IDEs-COF powder- and film-based sensors and the humidity testing are elaborated in Figs. S5–S8. However, the COF_X-DHTA_ powder-based humidity sensors have a low signal-to-noise ratio and a small signal amplitude over a wide RH range (0–74.9% RH). As a result, COF powders are incompatible with applications requiring ultra-sensitive humidity detection. The performance comparison between COF_X-DHTA_ powder- and film-based sensors can further elaborate the advantages of COF_X-DHTA_ films in sensing. Furthermore, we investigate the relationship between COF film thickness and sensing performance. The 1.5-COF_TAPB-DHTA_ film (108.9 μm) from the X-COF_TAPB-DHTA_ series with the μm-level exhibited the highest response values (Figs. S9 and S10). To exclude the interference of the complex ambient atmosphere, selectivity tests for different vapors (including water) were also performed. The COF_X-DHTA_ films have an intense response to water vapor, and the most outstanding selectivity is found in the case of the COF_TAPT-DHTA_ film with a strongly polar triazine structure (Fig. S11) [28]. On the other hand, the COF_TAPB-BPDA_ and COF_TAPB-PDA_ film displayed a negligible humidity response and a low signal-to-noise ratio. Therefore, it is confirmed that the more electron-rich structural functional monomer of the COF films generated a higher humidity sensing response value. The humidity response is defined here using Eq. (1):

1 $${\text{Response}}\,\% \, = \left( {\frac{{I_{{{\text{humidity}}}} - I_{{{\text{dry}}}} }}{{I_{{{\text{dry}}}} }}} \right) \times 100\%$$

where *I*_humidity_ and *I*_dry_ represent the current of the sensor under analyte vapors and dry compressed air, respectively.

## Results and Discussion

### Characterizations of COF Powders and Films

**Fig. 2:**
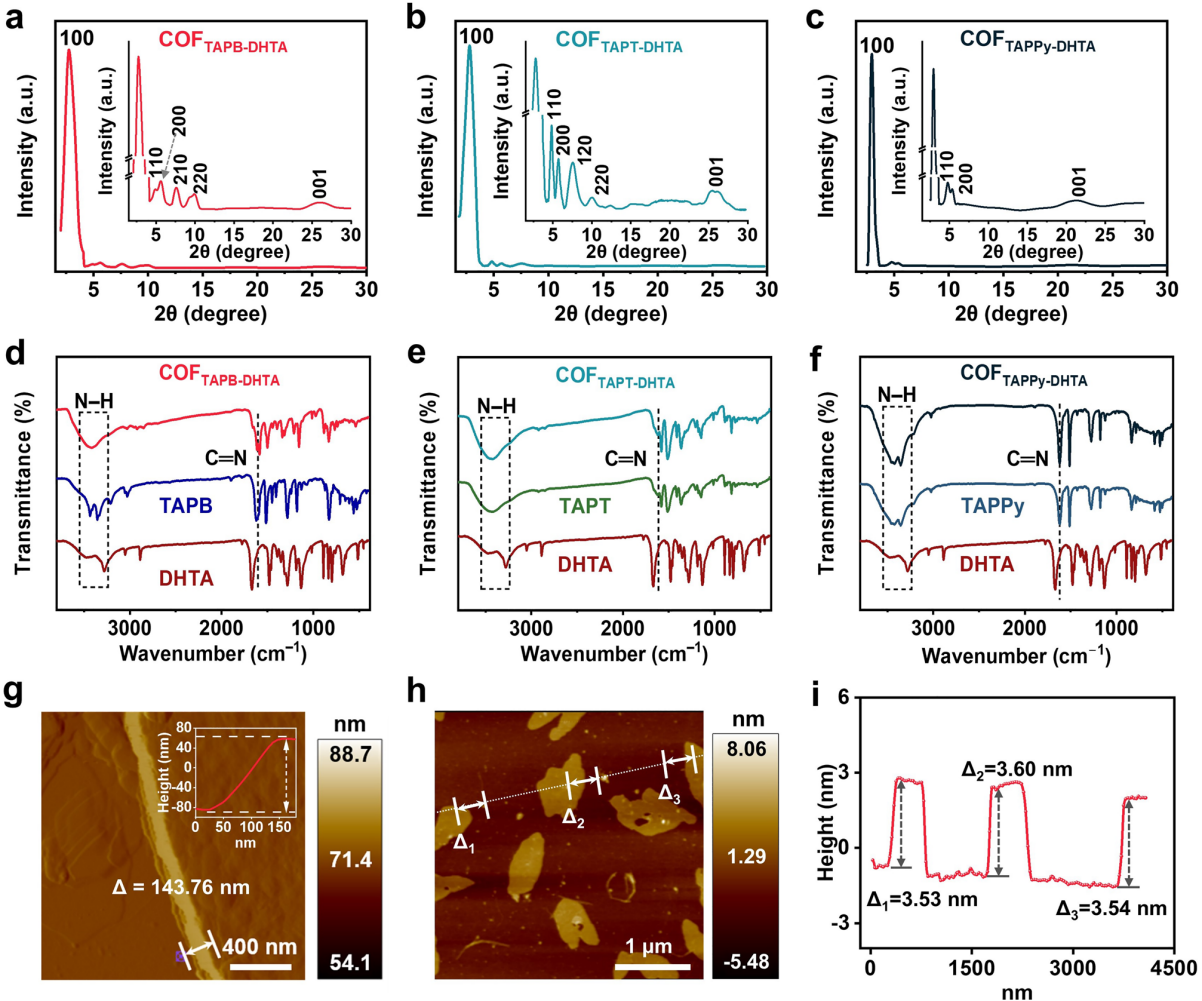
PXRD patterns of **a** COF_TAPB-DHTA_, **b** COF_TAPT-DHTA_, and **c** COF_TAPPy-DHTA_ film. FT-IR spectra of **d** COF_TAPB-DHTA_, **e** COF_TAPT-DHTA_, and **f** COF_TAPPy-DHTA_ film. **g** AFM height image of COF_TAPB-DHTA_ film. **h** AFM height image of COF_TAPB-DHTA_ film after ultrasound for 3 h.** i** Height profile along the white line in (**h**)

The powder X-ray diffraction (PXRD) patterns of COF films depicted in Fig. 2a–c contain some distinct reflections, indicating the acquisition of the predicted structure of COF films with high crystallinity. The features at 2.75°, 4.93°, 5.62°, 7.59°, and 9.90° are denoted as (100), (110), (200), (210), and (220) facets, respectively. Notably, there is a weak peak at the angle of 26.02°, corresponding to the (001) reflection of COF_TAPB-DHTA_ [17]. Comparing the experimental and simulated results of PXRD, we can determined that COF_TAPB-DHTA_ is in the AA stacking mode (Fig. S12). The COF_TAPT-DHTA_ and COF_TAPPy-DHTA_ also exhibit AA stacking similar to previously reported COF structures [26, 29]. Excluding the amorphous state in the contrast films that affect the sensing results, the sharp reflections of PXRD patterns were also obtained (Fig. S13). Furthermore, the stretching characteristic peak of C=N produced by imine condensation confirms the existing topology of COF films, which is about 1620 cm^−1^ in the fourier transform infrared spectroscopy (FT-IR) spectra. A new characteristic peak that appears at 3300–3500 cm^−1^ can be ascribed to the N–H stretching vibration of amine groups and the hydroxyl on the DHTA monomer (Figs. 2d–f and S14) [30]. To evaluate the chemical components of the prepared COF_X-DHTA_ films, XPS analysis was performed. The full XPS spectra of the COF_X-DHTA_ films indicate that only C, N, and O elements exist, and the peak positions of each element are very similar (Fig. S15). The prepared ultrathin COF films with nm-level thickness can facilitate clearer observation of the microscopic morphology of COF films. According to the atomic force microscopy (AFM) images, the thickness of the prepared ultrathin COF_TAPB-DHTA_ film with layer-by-layer self-assembly stacking at the interface is 143.8 nm (Fig. 2g) [31]. The stabilized nm-level COF_TAPB-DHTA_ film can also be delaminated to homogenous thinner sheets. The average thickness is approximately 3.56 nm by ultrasonication for 3 h in alcohol. Comparing the 3D topography and AFM height of the nm-level COF_X-DHTA_ films, it was found that the degree of uniform dispersion of COF_TAPB-DHTA_, COF_TAPT-DHTA_ and COF_TAPPy-DHTA_ films gradually decreases. As a result, there are variations in the stacking microstructure of the three samples, which might affect their capacity to sensors detect humidity (Figs. 2h and S16) [32]. The average thickness is approximately 3.56 nm by ultrasonication for 3 h in alcohol. Comparing the 3D topography and AFM height of the nm-level COF_X-DHTA_ films, it was found that the degree of uniform dispersion of COF_TAPB-DHTA_, COF_TAPT-DHTA_, and COF_TAPPy-DHTA_ films gradually decreases. As a result, there are variations in the stacking microstructure of the three samples, which might affect their capacity to sensors detect humidity (Figs. 2h and S16) [32].

**Fig. 3:**
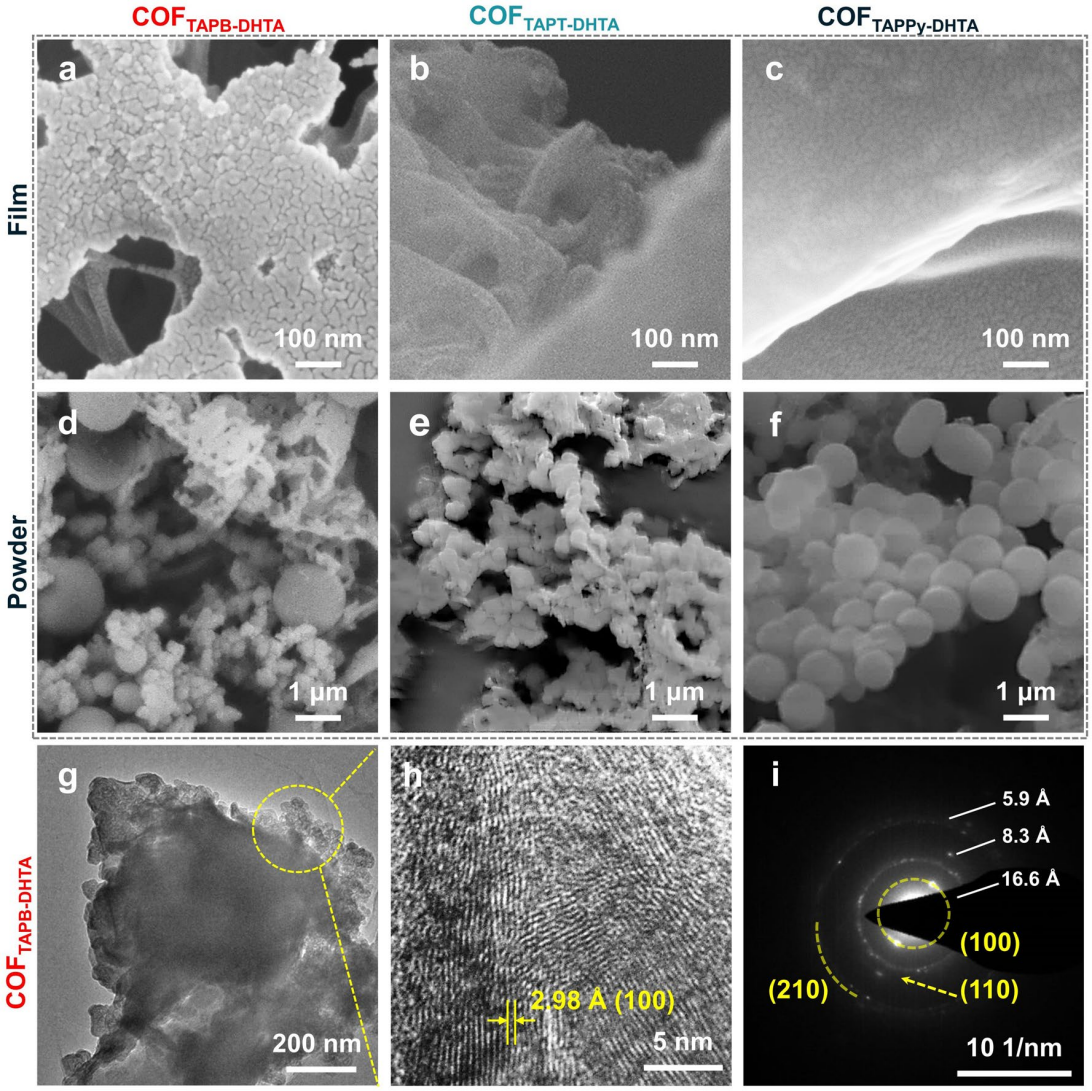
SEM images of **a** COF_TAPB-DHTA_, **b** COF_TAPT-DHTA_, and **c** COF_TAPPy-DHTA_ film. SEM images of **d** COF_TAPB-DHTA_, **e** COF_TAPT-DHTA_, and **f** COF_TAPPy-DHTA_ powder. **g** TEM, **h** HR-TEM and **i** SAED patterns of COF_TAPB-DHTA_

Scanning electron microscopy (SEM) images of COF films demonstrate the continuous plane accompanied by porous morphology on the surface (Figs. 3a–c and S17–S18). In contrast, the COF powders prepared by the solvothermal method possess a uniform spherical shape with disorderly stacked blocks (Fig. 3d–f). The clear lattice fringes can be observed in high-resolution transmission electron microscopy (HR-TEM) images of COF_TAPB-DHTA_ film, where the spacing is about 2.98 Å, corresponding to (100) facets (Fig. 3h) [33]. HR-TEM images and selected area electron diffraction (SAED) patterns of the COF_TAPB-DHTA_ film are consistent with the crystal structure provided by PXRD results (Fig. S19) [34]. The stacking, crystallinity and grain size of COFs will seriously interfere with the transition energies required for electron transfer during the sensing process [35]. Therefore, the long-range continuous porous COF films will accelerate charge transfer and provide a solid basis for favorable sensing performance [36].

The Brunauer–Emmett–Teller (BET) measurement result of μm-level COF films is shown in Fig. S20. The nitrogen sorption isotherm exhibits a type IVb isotherm with a hysteresis loop, confirming the mesoporous character of the COF_TAPB-DHTA_ and COF_TAPT-DHTA_ films (Fig. S20a, b). The equilibrium model of quenched solid density functional theory (QSDFT) analyzes COF_TAPB-DHTA_ film and yields a very narrow pore size distribution with a maximum of 2.28 nm (Fig. S20a) which agrees with the structural model in SEM and TEM images. COF_TAPB-DHTA_ film has the largest BET surface of 1143.18 ± 38.66 m^2^ g^−1^, with a cumulative pore volume of 0.63 ± 0.05 cm^3^ g^−1^. There are two peaks in the cumulative pore size volume of COF_TAPPy-DHTA_ film, which play an adsorption function at 2.34 and 32.23 nm, indicating the existence of a hierarchically porous structure (Fig. S20f). The alternating stacking of COF_TAPPy-DHTA_ film layers containing heterosporous frameworks may cause an even more pore volume reduction. DHTA hydrogen bonding can prevent a nucleophilic attack during the condensation reaction, resulting in COF_X-TAPB_ films with a controllable pore size (Figs. S20d, e and S21). In contrast, the pore size distribution of COF_TAPB-BPDA_ and COF_TAPB-PDA_ films without DHTA is more dispersed. COF_TAPB-BPDA_ and COF_TAPB-PDA_ films only have BET surface results of 666.26 ± 13.31 and 611.16 ± 6.49 m^2^ g^−1^, respectively (Fig. S21a, b).

### Humidity Detection and Applications Based on the IDEs-COF Film Sensors

**Fig. 4:**
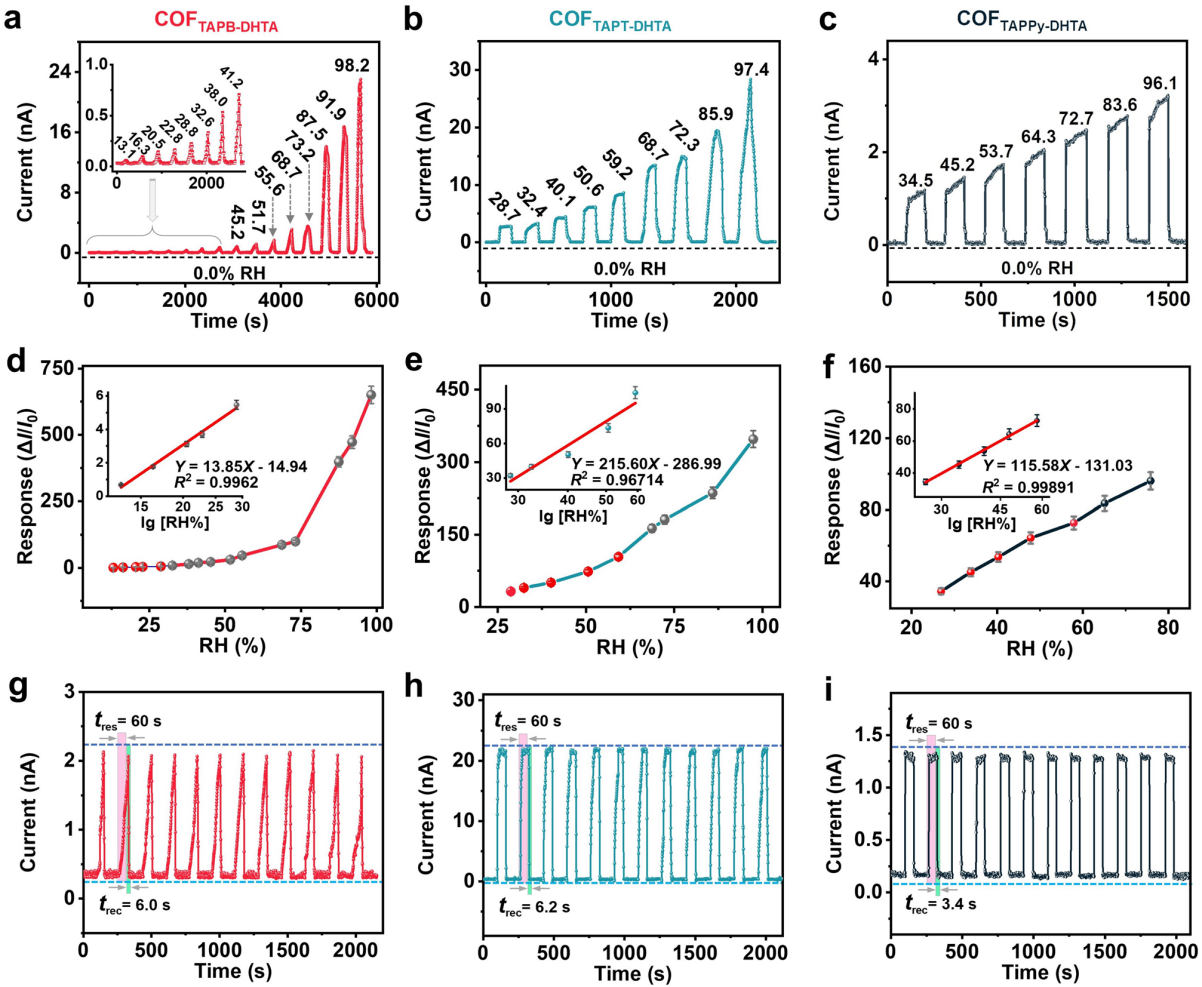
Dynamic response characteristic curves of **a** IDEs-COF_TAPB-DHTA_, **b** COF_TAPT-DHTA_, and **c** COF_TAPPy-DHTA_ film-based humidity sensors at different RH (Detection range: from dry air 0.0% to different RH). Response versus RH curve of **d** COF_TAPB-DHTA_, **e** COF_TAPT-DHTA_, and **f** COF_TAPPy-DHTA_ film (the inset shows the linear fit curve of response versus the RH logarithm). Response and recovery curves for 12 cycles of **g** COF_TAPB-DHTA_, **h** COF_TAPT-DHTA_, and **i** COF_TAPPy-DHTA_ film (Detection range: 41.7–76.2% RH)

Within the COF_X-DHTA_ films, the most sensitive film is COF_TAPB-DHTA_ with the lowest detection limit when using the normalizing control of water vapor exposure time. The real-time current response curve can still be observed when the relative humidity is under 50%. Within a 3.2% RH change (from 13.1 to 16.3%, the low RH), the response value increased by as much as 108.7% (inset in Fig. 4a). The IDEs-COF_TAPB-DHTA_ film-based sensor has a current amplitude enhancement of 390 times when the humidity ranges from 13.1 to 98.2% RH (Fig. 4a). Such an ultra-high sensitivity performance of the COF film-based sensor is critical for the perturbation of human-related humidity detection [37].

As contrasted with COF_TAPB-DHTA_, COF_TAPT-DHTA_ and COF_TAPPy-DHTA_ films were less sensitive with higher detection limits (Fig. 4b, c). A logarithmic fit for the response and RH from the lowest detection capability of the COF_X-DHTA_ film-based sensor to 60% RH (atmospheric environment in sunny weather) shows a linear relationship for all films (Fig. 4d–f insets). The fact that COF films can be designed at the molecular level, shows an excellent linear response at low humidity. As for COF_TAPB-DHTA_ film, the theoretical detection limit can reach 7%, which is far better than the detection ability of traditional humidity sensors at RT (Fig. 4d inset) [38]. It is clarified that by fine-tuning the ligand monomers and functional groups of the COF topology, we can achieve the precise design of functional structural units adapted to multiple application scenarios with high sensitivity, wide range, fast response, and high linear relationship. To evaluate the humidity monitoring stability of the COF films, we performed the cycle stability test in the atmosphere of ambient humidity. Even after 12 cycles, there is a negligible change in the current output characteristic curve, suggesting the COF_X-DHTA_ films are stable (Fig. 4g–i). When the IDEs-COF film-based sensor is exposed to a high-humidity atmosphere (76.2% RH) for 60 s, it recovers only in 3.4 s on average (Fig. 4i). Long-term sensing response tests demonstrate the structural stability of the COF_TAPB-DHTA_ film (Fig. S22). The humidity test for the same COF_TAPB-DHTA_ film was repeated every two months under the same conditions. After ten months, the COF film-based humidity sensor retained 87.7% of its original response value.

**Fig. 5:**
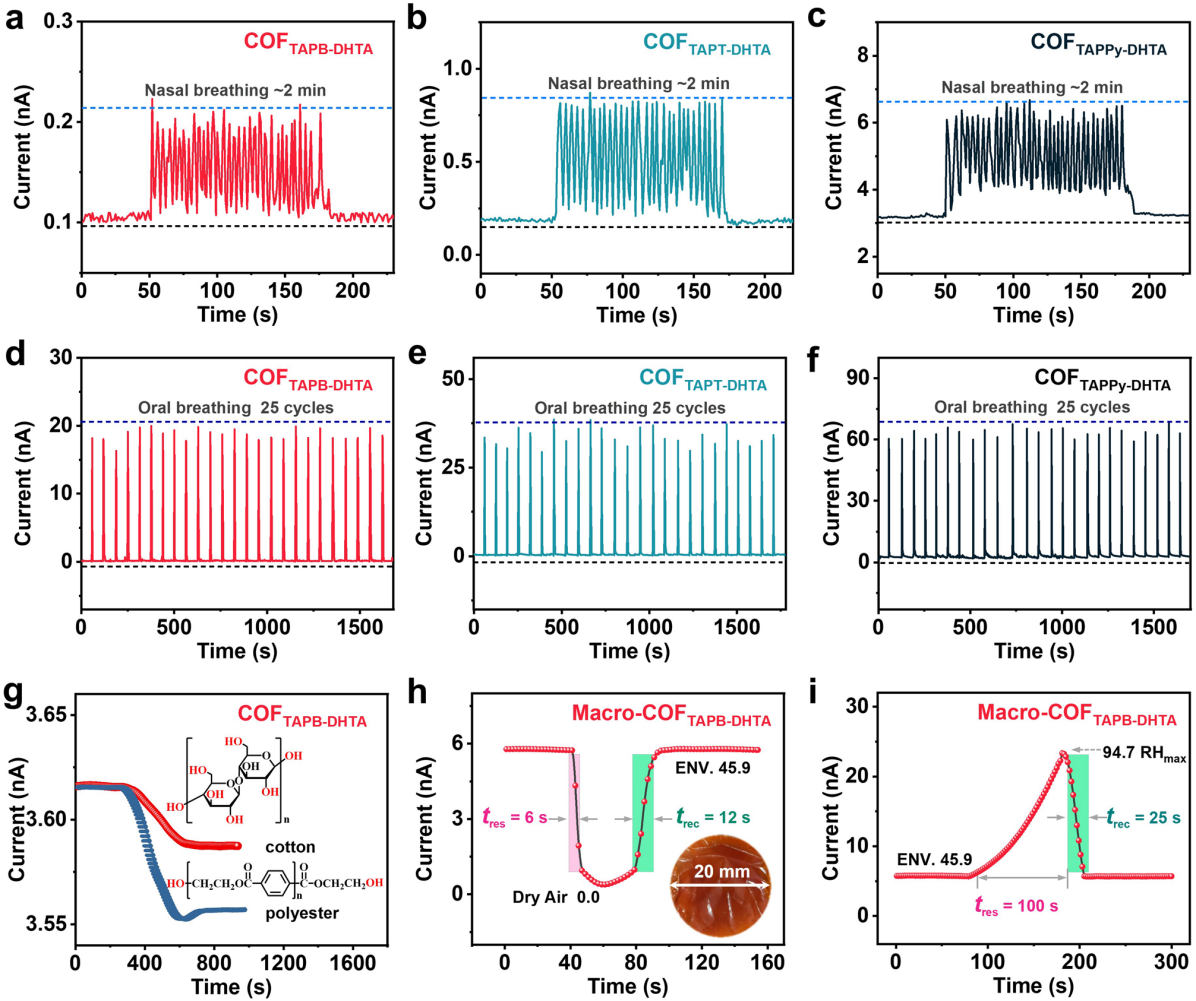
Nasal breathing monitoring curves of **a** IDEs-COF_TAPB-DHTA_, **b** COF_TAPT-DHTA_, and **c** COF_TAPPy-DHTA_ film-based humidity sensor (the environment RH performed at 52.3%). Oral breathing monitoring curves of **d** IDEs-COF_TAPB-DHTA_, **e** COF_TAPT-DHTA_, and **f** COF_TAPPy-DHTA_ film-based humidity sensor (the environment RH performed at 31.7%). **g** Real-time current curve of the IDEs-COF_TAPB-DHTA_ film-based humidity sensor simulates the air permeability of clothing fabrics. Real-time dynamic humidity monitoring directly using a Macro-COF_TAPB-DHTA_ film-based humidity sensor on the glass slide detection range from **h** dry air to environment RH (0.0–45.9%) and **i** environment to maximum RH (45.9–94.7%)

Benefiting from the fast response and short interval periods of the COF film-based humidity sensor, we investigated its potential application for monitoring human breath rate. The IDEs-COF_X-DHTA_ was used to detect two of these breathing patterns of volunteer-I, namely oral and nasal breathing. Previous humidity monitoring reports indicate that the RH of oral breathing is higher than that of nasal due to the presence of saliva in the mouth [39]. As shown in Fig. 5a–f, our detection yields the same result, the oral breathing monitoring signal current value is greater than the nasal. Volunteer-I must rest for 1 min before each test and wear a mask with a breathing valve (Fig. S23). The IDEs-COF_X-DHTA_ film-based humidity sensor is embedded in the mask to ensure that the data of each test is relatively objective and accurate. The nasal breathing monitored for 2 min shows that the response and recovery times of the IDEs-COF_TAPB-DHTA_ film-based sensor are 0.4 and 1 s, respectively (Fig. 5a). Likewise, the real-time current signal curve increased significantly in the oral breathing across 25 cycles performed by volunteer-I with a stable amplitude as shown in Fig. 5d–f. As we know, the average humidity of the transient airflow carried by human breath is generally over 90% RH [39]. The sensing results from Fig. 5a–f indicated that the COF_X-DHTA_ film-based sensor maintains good sensing performance even at a high RH of over 90%, highlighting its potential for human respiration monitoring with good stability.

Remarkably, it is meaningful to evaluate the breathability of clothing in direct contact with the skin, because the skin plays a key role in human life activities, such as the detection of cotton and polyester fabrics' air permeability [41]. Evaporating normal saline at 36 ± 0.5 °C is used as a simulation of sweat on the surface of human skin, and the distance between the container neck and the IDEs-COF_TAPB-DHTA_ film-based sensor is set at 20 mm (Fig. S24). After stabilizing for 400 s, the conical flask was completely covered with fabrics, and real-time dynamic current curves were observed (Fig. 5g). The ventilation effect of pure cotton fiber is found to be significantly better than that of polyester and tends to stabilize after about 350 s (red curve in Fig. 5g). As shown in the chemical structural formula of cellulose with abundant hydroxyl groups that can form hydrogen bonds and assist in transporting water molecules. Interestingly, polyester fibers with a highly crystalline molecular chain structure quickly hindered the evaporation of “sweat”. Due to the accumulation of water molecules on the surface (after about 200 s), the capillary core transfer occurs on the polyester fiber, allowing a small number of water molecules to overflow the surface. Hence, the detection current increases slightly (blue curve in Fig. 5g) before stabilizing. In addition, a series of interference tests on the IDE-COF-based sensor, the conditions of the experiments including a mixed gas atmosphere (Fig. S25), light illumination under different wavelengths (Fig. S26), variable RT (Fig. S27a–c), and the hysteresis response (Figs. S28 and S29) were conducted. And all testing results reveal that the sensitivity and stability of the COF_X-DHTA_ film-based sensor are excellent for humidity.

Moreover, we conducted a humidity sensing test using the Macro-COF_TAPB-DHTA_ film (*d* = 20 mm) under the glass substrate. The dynamic response curves of the Macro-COF_TAPB-DHTA_ film-based sensor at RT with a detection range from dry air to environment RH (0.0–45.9%) and then to max RH (45.9–94.7%), respectively (Fig. 5h, i). The recovery time is a little longer compared to the IDEs-COF_X-DHTA_ film-based sensors. The main reason for this phenomenon is the accumulation of water molecules on the macroscopic film, which leads to a longer recovery time under the same atmospheric environment. A comparison of the humidity detection performance of the COF_TAPB-DHTA_ film-based sensor with previously reported studies are shown in Table S1. It can be seen that our COF film material exhibits a comprehensive sensing capability. The humidity sensor based on COF_TAPB-DHTA_ film's superior sensing performance is expected to spark new ideas for monitoring RH in human metabolite detection.

### Humidity Sensing Mechanism

**Fig. 6:**
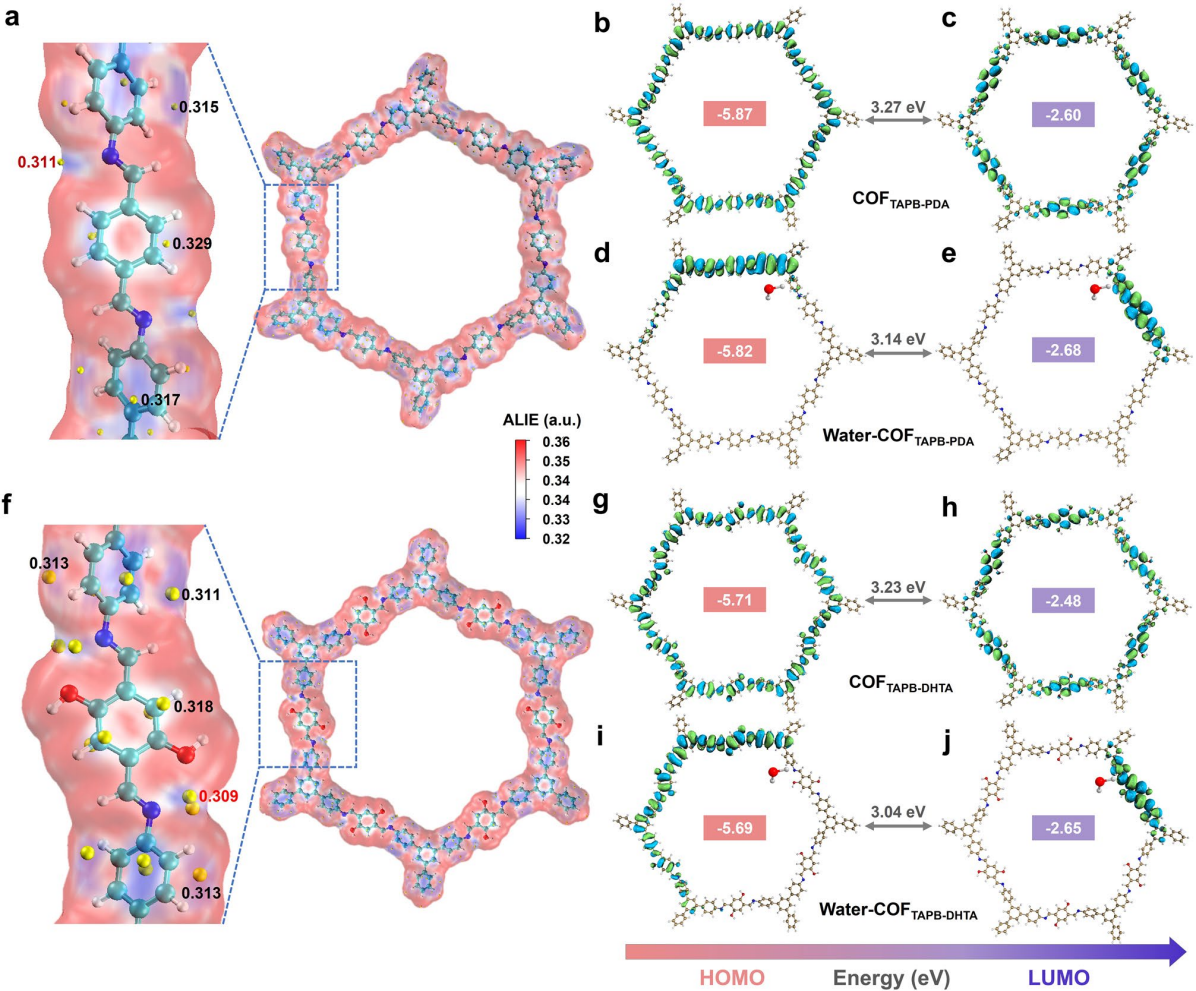
**a** ALIE distribution on the surface of COF_TAPB-PDA_ film. **b** HOMO and **c** LUMO of COF_TAPB-PDA_ film. **d** W-HOMO and **e** W-LUMO of Water-COF_TAPB-PDA_ is formed by absorbing water molecules of COF_TAPB-PDA_ film. **f** ALIE distribution on the surface of COF_TAPB-DHTA_ film. **g** HOMO and **h** LUMO of COF_TAPB-DHTA_ film. **i** W-HOMO and **j** W-LUMO of Water-COF_TAPB-DHTA_ formed by absorbing a water molecule of COF_TAPB-DHTA_ film

Numerous hydroxyl functional groups bring in the hydrophilic properties of the COF films measured by the contact angle, which is approximately 58° (Fig. S30). The higher contact area facilitates the charge transfer between COF_X-DHTA_ films and water molecules, resulting in a faster response [42]. Based on DFT calculations and in situ Raman spectra, the sensing mechanism of COF films for humidity was discussed. The 4.2.1 ORCA software was used for all calculations, and the basis was B3LYP/6–31 + G(d) with the D3BJ correction function. The data processing and image rendering was executed by Multiwfn 3.8.0 and VMD 1.9.3 [43–45]. The average local ionization energy (ALIE) distribution provides accurate and reliable analysis regarding the reaction sites and binding mode (Figs. 6 and S31). It can be seen that the ALIE minimum points of both COF_TAPB-PDA_ and COF_TAPB-DHTA_ are distributed near the nitrogen atoms of the imine bond. When water molecules are present, the N atom acts as the active site, providing the least amount of electronic constraints to form the imino/*cis*-ketoenamine isomers. Inversely, the ALIE minimum site of COF_TAPT-DHTA_ is located at 0.33 a.u. position on the benzene ring. It was attributed to the COF_TAPT-DHTA_ with an N-rich triazine structure providing strong electron-absorbing ability, and framework equilibrium electron rearrangement of the neighboring benzene ring as a more suitable active site [46]. In particular, the COF_TAPB-DHTA_ has the lowest ALIE minimum of 0.309 a.u. compared to the other COFs. In terms of thermodynamics, it is highly favorable for water molecules to attack the imine bond in COF_TAPB-DHTA_ and lead to reciprocal isomerization (Fig. 6a, f). To further reveal the humidity sensing mechanism, the highest occupied molecular orbital (HOMO) and the lowest unoccupied molecular orbital (LUMO) of COF_TAPB-PDA_, and COF_X-DHTA_ which have demonstrated humidity-sensitive, as well as those of Water-COF_TAPB-PDA_ and Water-COF_X-DHTA_ upon water adsorption were also investigated. The energy arrangement of the donors/acceptors is pivotal for achieving an efficient driving force for separating initially generated excitons. Following this principle, the HOMO and LUMO of COF films should be delocalized and located on different nodes that serve as donors/acceptors [25].

Furthermore, the hydroxyl group of DHTA supplies additional electrons, increasing the reactivity of the COF_TAPB-DHTA_ film. The polarity of the hydroxyl group induces enhanced van der Waals force interactions between the layers, and the denser stacking of COF_TAPB-DHTA_ can contribute to the transport of water molecules (Fig. S32). The W-HOMO of COF_TAPB-PDA_ is distributed around the benzene ring and cannot induce structural heterogeneity (Fig. 6d). However, the W-HOMO and W-LUMO configurations of Water-COF_TAPB-DHTA_ film show a more delocalized level on the whole framework, and it is beneficial for improving carrier mobility (Fig. 6i, j) [47]. The entry of the water molecule leads to the iminol/*cis*-ketoenamine isomerization and induces a HOMO destabilization via conjugation along the imine linkers, which strengthens the donating ability of the imine bonds and reduces the energy gap *E*_g_. Accordingly, the narrower *E*_g_ is obtained for Water-COF_X-DHTA_, suggesting the enhanced electron conductivity, which conforms to the collected real-time current value being positively correlated with the RH [48]. Comparing the *E*_g_ of the COF_X-DHTA_ film and the difference after adsorbing water molecule, where COF_TAPB-DHTA_ has the largest Δ*E*_g_ is − 0.19 eV, followed by COF_TAPPy-DHTA_ with − 0.13 eV (Table S2). Moreover, the weak interaction allows the COF_TAPB-DHTA_ film to rapidly perform the adsorption/desorption process at RT to achieve highly sensitive and reversible humidity sensing [49]. It is also worth mentioning that the triazine structure of COF_TAPT-DHTA_ makes the Δ*E*_g_ exhibit only − 0.044 eV. The extremely low Δ*E*_g_ allows COF_TAPT-DHTA_ to have a fast response value boost, but it also shows a slight perturbation in the light response test (Fig. S26). Analogous results are shown in the UV–Vis and UPS test spectra, as shown in Figs. S33–S35.

**Fig. 7:**
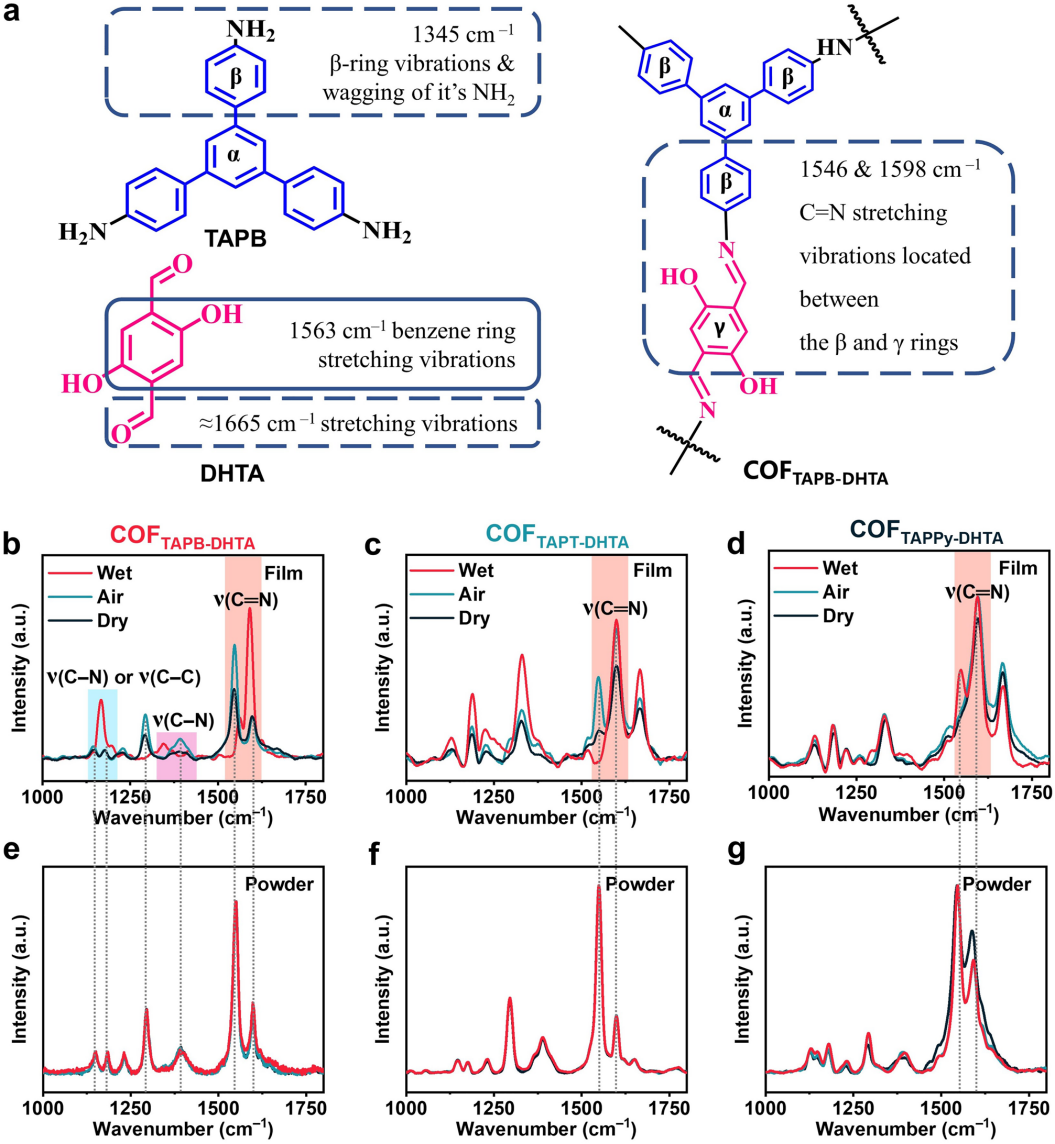
**a** Raman shift assignment of COF_TAPB-DHTA_. In situ Raman of **b** spectra COF_TAPB-DHTA_,** c** COF_TAPT-DHTA_, and **d** COF_TAPPy-DHTA_ film. In situ Raman spectra of **e** COF_TAPB-DHTA_, **f** COF_TAPT-DHTA_, and **g** COF_TAPPy-DHTA_ powder

Subsequently, we carried out in situ Raman under different RH conditions (including dry, atmosphere, and wet vapor are 0, 45%, and 90% RH, respectively) with a 532 nm laser (Figs. 7 and S36). As shown in Fig. 7a and Table S3, the Raman shifts of the COF_X-DHTA_ skeleton are assigned to typical vibrations of different groups. All of the COF films and powders have distinct characteristic peaks around 1600 cm^−1^, corresponding to the vibrational modes of (–C=N–) bonds, indicating that the skeleton structure of COF films may be maintained after humidity sensing. The aldehyde group (C=O) bond of DHTA stretching vibration is around 1665 cm^−1^ with a weak signal, which is caused by the photoluminescence phenomenon [50]. As the aforementioned humidity sensing test findings show, there is no shift in the Raman peaks of COF_TAPB-BPDA_ and COF_TAPB-PDA_ powders and films for the comparison samples (Fig. S36). The characteristic peak positions of COF_X-DHTA_ powders scarcely shift under any conditions (Fig. 7e−g).

The tautomerism generated by water molecules on the outermost surface alone is insufficient to produce a significant deflection peak of the COF_X-DHTA_ powders [51]. Unlike the powder, COF film provides active sites and a conjugated conductive platform for water molecules through a large-scale and precise structural design, realizing outstanding humidity sensing performance. Under dry and air atmosphere conditions belonging to the low humidity (black and blue curves in Fig. 7b, c, < 50% RH), the intensity difference of characteristic peaks can be observed. However, with increasing RH, the band positions shifted sharply with the new torsional and stretching vibrations generated due to the tautomer induced by water molecules (red curves in Fig. 7b, c, 94.3% RH) [52]. The characteristic peaks of the COF_X-DHTA_ films are around 1550 cm^−1^ (aromatic υ(CC) aromatic ring chain vibrations) and 1590 cm^−1^ (V(C=N) strong), representing imine bond stretching vibrations located between the *β* and *γ* rings drastically changed [53]. It implied that water molecules bind to the *β*-site stretching vibration close to the host framework of TAPB, proving the active site is indeed the N atoms [54]. The COF_TAPT-DHTA_ and COF_TAPPy-DHTA_ film have more prominent peaks at this position because they are increasingly electron-rich monomers than the TAPB of COF_TAPB-DHTA_ film and thus exhibit higher response values in humidity sensing tests (Fig. 7c, d). Especially, two bands at 1143.98 and 1178.54 cm^−1^ in COF_TAPB-DHTA_ film can be assigned to *v*(C–N) or (C–C) aromatic under the low RH while turning into a single peak after water vapor treatment at high RH, respectively (the light blue square in Fig. 7b) [55]. In addition, the band at 1392.07 cm^−1^ (dry) shifts to 1344.93 cm^−1^ (wet) assigns to the *v*(C–N) of COF_TAPB-DHTA_ film vibration (pink square in Fig. 7b) [56]. It is also confirmed that COF_TAPB-DHTA_ film can generate two stretching vibration effects induced by *cis-Keto* imine tautomerism (Fig. 7b) [19]. It reasonably explains that the humidity sensor based on COF_TAPB-DHTA_ film has a more sensitive and quantitative detection ability than other sensors. In view of the above arguments, based on the above arguments, the imine groups in COF films acted as dual-active sites for humidity sensing, inducing an intrinsic enhanced mechanism of reversible protonated tautomerism via water molecule hydrogen bonding is summarized in Fig. S37.

## Conclusions

In this work, we presented humidity-responsive COF-based films for monitoring human respiration. The COF_X-DHTA_ films exhibited excellent crystallinity and reversible tautomerism, resulting in intrinsic resistance variations while maintaining high anti-interference and long-term sensitivity. The structure–property relationship between imine reversible isomerization and ultra-sensitive sensing of COF_TAPB-DHTA_ film has been established by analyzing the functional group species, bonding types, minimum polarity point, and N content of COF films with the support of in situ Raman spectroscopy measurement and DFT calculations. A respiration monitoring experiment was performed to generate a respiration frequency floating signal with a response/recovery time of 0.4/1 s. Additionally, this COF_X-DHTA_ film can be directly used as a sensor for humidity sensing, without any additional processing steps. This work utilizes a facile synthesis method to fabricate uniform and stable COF films, which have the potential for applications of flexible electronics and wearable devices in quantitative human respiratory monitoring. These findings are crucial for the development of new sensing materials and devices in the field of respiratory monitoring.

### Supplementary Information

Below is the link to the electronic supplementary material.

Supplementary file1 (PDF 3555 KB)
