# Supplementary material for: Outstanding Humidity Chemiresistors Based on Imine-Linked Covalent Organic Framework Films for Human Respiration Monitoring
Source: Nanomicro Lett. 2023 Jun 7;15:149. doi: 10.1007/s40820-023-01107-4 (PMC10247948; doi:10.1007/s40820-023-01107-4)
Supplement: Supplementary file 1 — Supplementary file1 (PDF 3555 KB) [file 40820_2023_1107_MOESM1_ESM.pdf]

Supporting Information for

Outstanding Humidity Chemiresistors Based on Imine-Linked Covalent Organic Framework Films for Human Respiration Monitoring

Xiyu Chen[1], Lingwei Kong[1], Jaafar Mehrez[1], Chao Fan[1], Wenjing Quan[1], Yongwei Zhang[1], Min Zeng[1,]*, Jianhua Yang[1], Nantao Hu[1], Yanjie Su[1], Hao Wei[1], and Zhi Yang[1,]*

[1]Key Laboratory of Thin Film and Microfabrication (Ministry of Education), Department of Micro/Nano Electronics, School of Electronic Information and Electrical Engineering, Shanghai Jiao Tong University, Shanghai 200240, P. R. China

*Corresponding authors. E-mail: minzeng@sjtu.edu.cn (Min Zeng), zhiyang@sjtu.edu.cn (Zhi Yang)

S1 Materials, Preparation Methods of COFs Films and Powders

S1.1 Materials

All the monomers used in the synthesis of COFs films and powders, namely 1,3,5-tris(4-aminophenyl)benzene (TAPB), 1,3,5-tris-(4-aminophenyl)triazine (TAPT), 1,3,6,8-tetrakis(4-aminophenyl)pyrene (TAPPy), 2,5-dihydroxyterethaldehyde (DHTA), 1,4-phthalaldehyde (PDA), and 4,4'-biphenyldicarboxaldehyde (BPDA), were purchased from Changchun Jilin Zhongke Technology Co., Ltd., purity≥98%, with no further processing. The involved solvents include acetic acid (Aladdin, ≥99%), tridecane (Macklin, 98%), 1,4-dioxane (Aladdin, ≥99%), mesitylene (Acros Organics, 98%), and deionized water (18.2 MΩ·cm).

Commercially purchased solvents for testing sensor selectivity were used directly. To obtain solvent vapors, a bubbling technique was adopted, including methanol (Aladdin, ≥99%), tetrahydrofuran (THF, Aladdin, ≥99%), dichloromethane (DCM, OKA, 99.9%), acetone (OKA, ≥99.7%), isopropyl alcohol (2-Propanol, Aladdin, ≥99%), n-butanol (Aladdin, ≥99.4%), ethanol (Aladdin, ≥99.8%), ethylene glycol (Aladdin, >99%).

S1.2 Preparation Methods of Different COF Films and Powders and Apparatus

S1.2.1 Preparation of Different COF Films

The two kinds of monomers (TAPB and DHTA) were added in a 1:2 molar ratio and the solution concentration was 1 mmol mL^{-1}. DHTA was dissolved in the oil phase (tridecane), and TAPB was dissolved in the aqueous phase (6 M acetic acid solution). Removed the undissolved monomers after ultrasonic dispersion, slowly mix the uniform acidic water phase and oil phase in a clean container, and control the interface distance from the bottom of the container by 1 cm. After a reaction of 72 h, free-standing thin $COF_{TAPB-DHTA}$ film was successfully fabricated at the oil/water interface. The COF films were repeatedly washed in methanol and acetone to remove unreacted monomers and solvent residues. A series of films with different thicknesses were prepared. The mass of the TAPB monomer was kept constant, and the mass of DHTA monomer was controlled to be 0.2, 0.5, 1.0, 1.5, and 2.0 mg, named 0.2-$COF_{TAPB-DHTA}$, 0.5-$COF_{TAPB-DHTA}$, 1.0-$COF_{TAPB-DHTA}$, 1.5-$COF_{TAPB-DHTA}$, and 2.0-$COF_{TAPB-DHTA}$ film, respectively. The 1.5-$COF_{TAPB-DHTA}$ film described throughout the text will be abbreviated as $COF_{TAPB-DHTA}$ film without discussing thickness, and will not be repeated later.

S1.2.2 Preparation of Different COF Powders

COFs powders were prepared following the previously reported solvothermal method [16].The preparation of $COF_{TAPB\text{-}DHTA}$ powder is taken as an example to elaborate. Other powders were prepared according to the same procedure. TAPB (21.5 mg, 0.06 mmol), DHTA (15.1 mg, 0.09 mmol), controlled the reactant molar ratio at 2:3. Ultrasonic dispersion in 2 mL 1,4-dioxane and mesitylene mixed solvent (volume ratio 4:1). Added 0.2 mL of 4 M acetic acid as catalyst. After that, the mixture was sealed and heated to 120 °C for 72 h without stirring. Thorough washing of the product with THF and methanol yields COF powders.

S1.2.3 Preparation of IDEs -COF Film-based Humidity Sensors

The COF films were repeatedly washed with methanol and acetone to remove the insufficiently reacted coordination monomers and residual oil phase. A small piece of COF film was selected from a large scale and attached to the surface of the IDEs by *in situ* transfer at the solution interface. The IDEs require cleaning and hydrophilic treatment. It can also be observed from the SEM images that the COF film and the IDEs were flat and tightly combined, providing sufficient guarantee for the stability of the sensing signal. The COF powder-based humidity sensor is prepared by the traditional drop coating method, and the detailed operation steps are shown in the Supporting Information.

S1.2.4 Instrumental Characterization

The morphology and crystalline structure of the obtained samples were examined by field-emission SEM (FE-SEM, Ultra plus, Carl Zeiss, Germany), HR-TEM (HR-TEM, JEM-2100, JEOL, Japan), and PXRD (D8 Advance, Bruker, Germany) with Cu Kα source (λ = 0.15418 nm). AFM was collected with an MFP-3D, and post-processing of the AFM data has been done using the Asylum Research software, scanning area of 2 μm×2 μm for $COF_{TAPB\text{-}DHTA}$ film and a scanning area of 4×4 μm for $COF_{TAPB\text{-}DHTA}$ film after ultrasound for 3 h. FT-IR spectra were recorded using Thermo Scientific Nicolet 6700 FT-IR spectrometer. The specific surface area was estimated by the Brunauer-Emmett-Teller (BET, Autosorb-iQ, Quantachrome, USA), and the pore size was calculated by the Barrett-Joyner-Halenda (BJH) method by measuring the N_2 adsorption-desorption isothermals at 77 K. The absorption spectra and band gap were performed by the ultraviolet-visible diffuse reflectance spectra (UV-Vis DRS, EV300, US). The valence band (VB) spectra were measured with a monochromatic He I light source (21.2 eV) and a VG Scienta R4000 analyzer. A sample bias of -5 V was applied to observe the secondary electron cutoff (SEC). The work function (Φ) can be determined by the difference between the photon energy and the binding energy of the secondary cutoff edge. Ultra Depth of Field 3D Microscope System (VHX-5000) to measure film thickness. Contact angle measurements were taken in Kruss Drop Shape Analyzer–DSA100 instrument. The Raman spectrum was recorded on a Raman spectrometer (Renishaw inVia) operating with a 532 nm laser. In particular, *in situ* Raman test of this work, COF films and powders were stabilized by exposing them to a solvent vapor environment for 2 min. Then, the bubbling method was used to simulate the humidity-sensing environment throughout the test to further understand the COF sensing process.

S2 Supplementary Figures and Tables

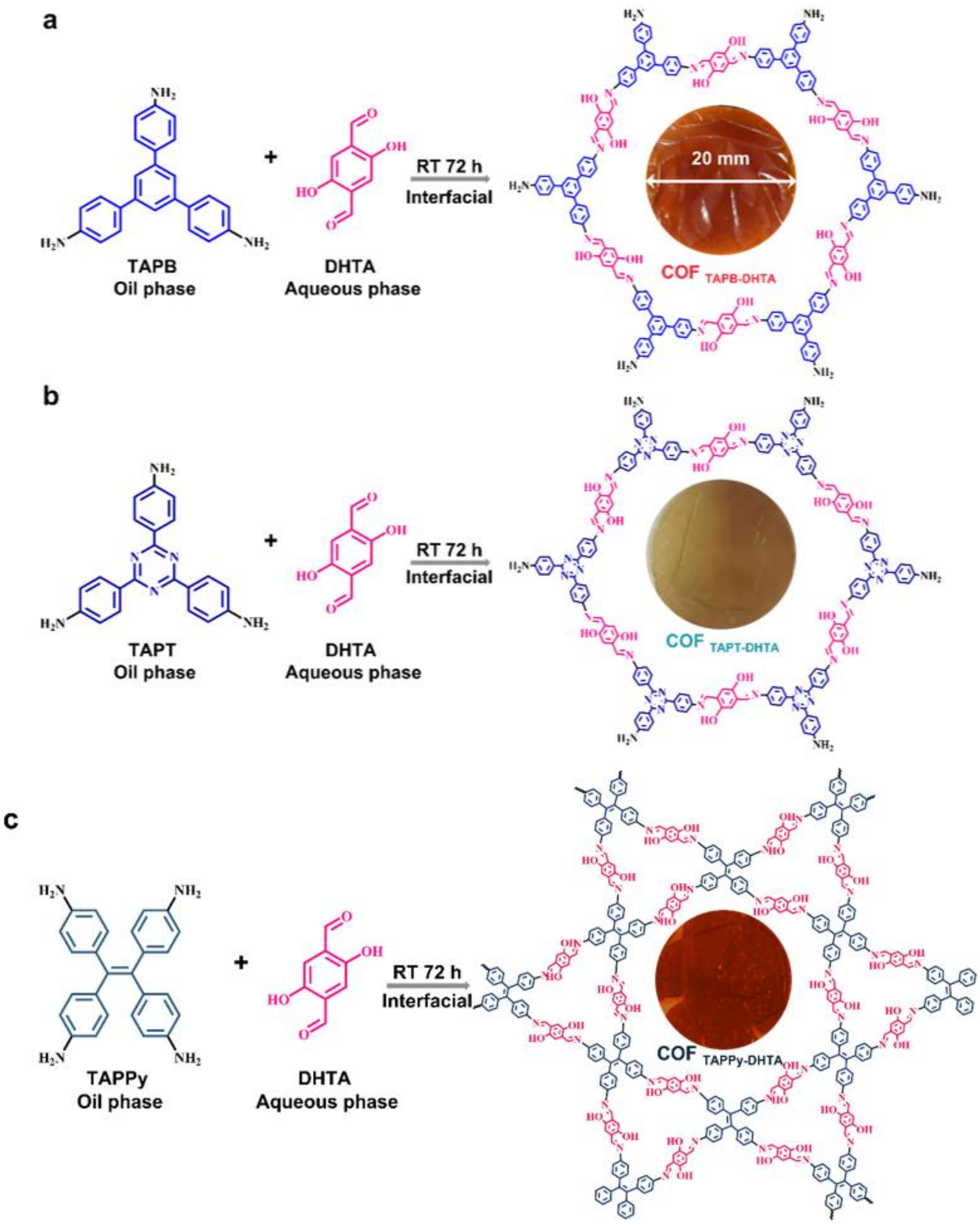

Fig. S1 a The chemical structure of the coordinating monomers of the $COF_{TAPB\text{-}DHTA}$, **b** $COF_{TAPT\text{-}DHTA}$, and **c** $COF_{TAPPy\text{-}DHTA}$ which has the humidity performance. The inserts are the optical photos of $COF_{X\text{-}DHTA}$ films

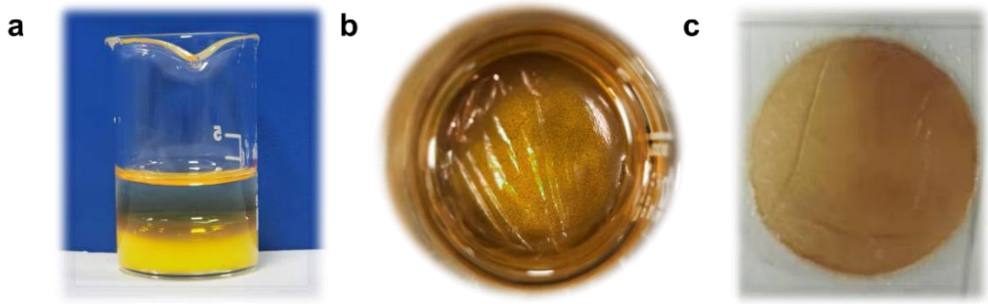

Fig. S2 a Side view and **b** top view of COF film fabricated at the oil/water interface. **c** After washing, the COF film was transferred to a glass slide

As for the morphology of these samples, contrast films $COF_{TAPT-DHTA}$, $COF_{TAPPy-DHTA}$, $COF_{TAPB-BPDA}$, and $COF_{TAPB-PDA}$ were also synthesized according to a similar process. The topology and monomer synthesis of several COF films are shown in Figs. S3 and S4.

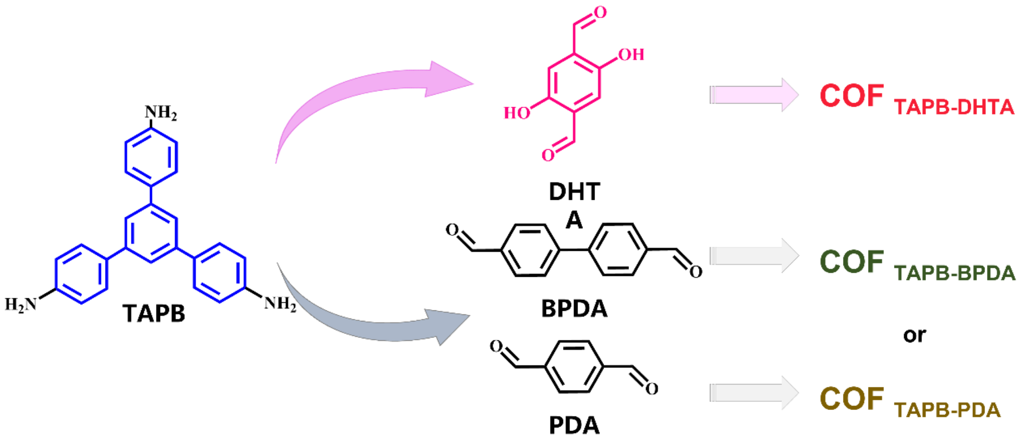

Fig. S3 Chemical structure of the coordinating monomers of the comparative samples $COF_{TAPB-BPDA}$ and $COF_{TAPB-PDA}$

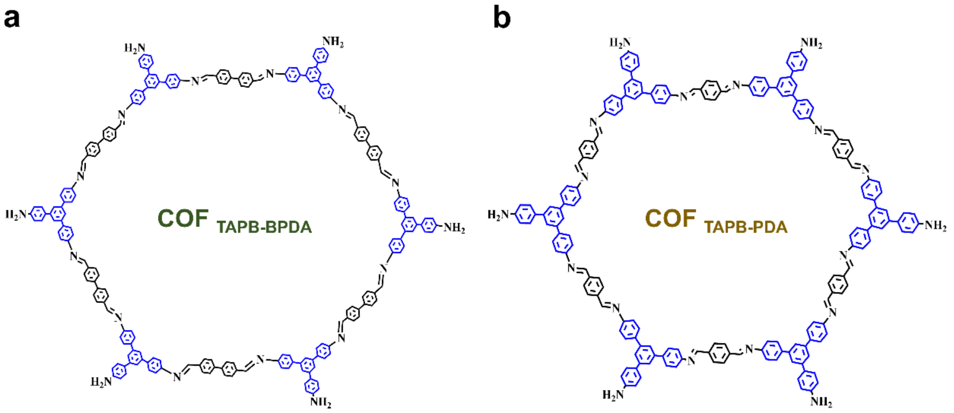

Fig. S4 a Topology of $COF_{TAPB-BPDA}$. **b** $COF_{TAPB-PDA}$

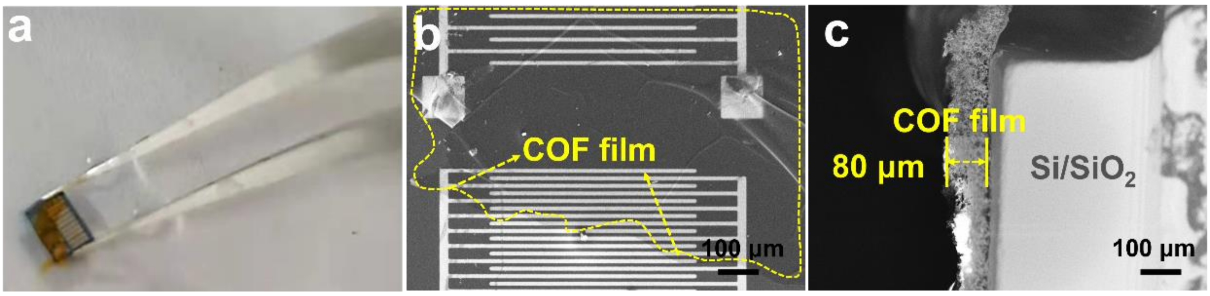

Fig. S5 a The photographs of *in situ* transfer process of the COF films to the IDEs at the solution interface. **b** SEM images front view and **c** cross-sectional view of the COF film attached to the surface of the IDEs

Schematic diagram of the humidity sensor measurement system (Fig. S6). By bubbling dry compressed air as the carrier gas in water and adjusting the mass flow ratio of dry gas and humidified gas, a stable relative humidity (RH) condition in the range from 7.4% to 98.6% can be obtained. The total gas flow for both channels was precisely controlled by a mass flow controller (MFC) at a constant rate of 200 standard cubic centimeters per minute (sccm). To ensure the measurement data is accurate, the remote monitoring temperature and humidity meter are placed in the test chamber for real-time monitoring. The actual relative humidity is

calibrated by a high-precision hygrometer (humidity accuracy: 3% RH, humidity resolution: 0.3% RH, BLE MAC: D6C93544DDFB, Chengdu Jiali Technology Co., Ltd., China).

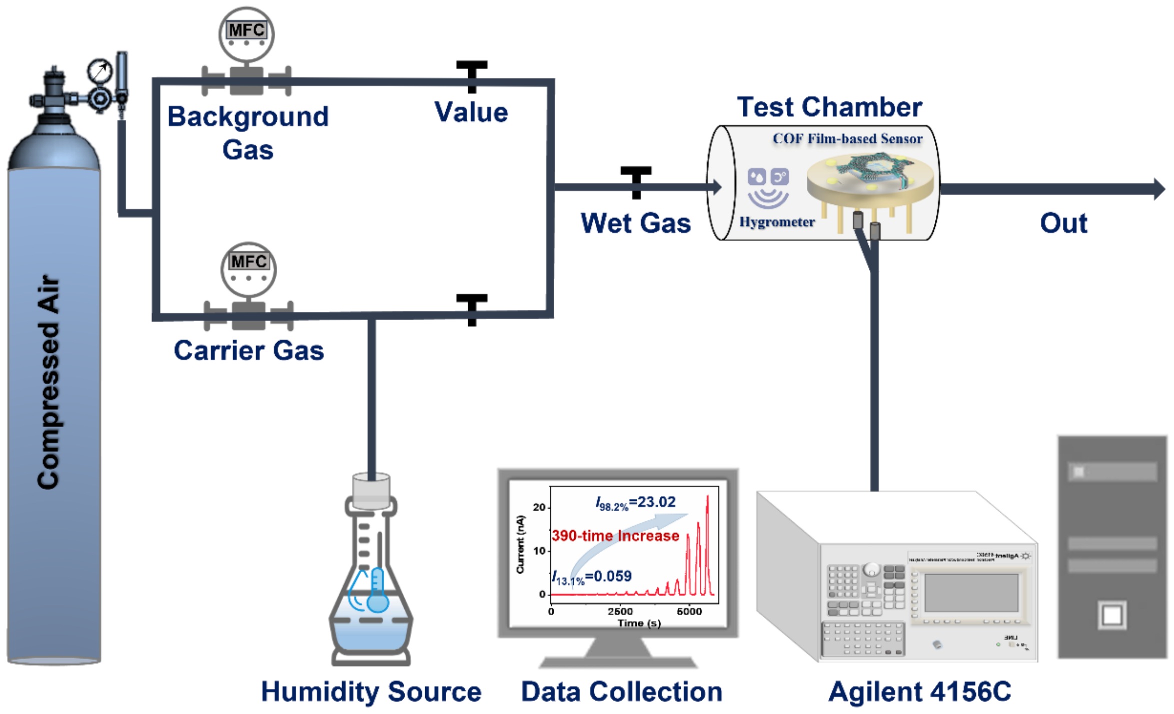

Fig. S6 Schematic diagram of the humidity sensor measurement system

To evaluate the humidity performance of COF film sensors, the real-time dynamic current curves were obtained by controlling the IDEs-COF film-based sensors in a humidity chamber for a specific time and recovering it in a dry air atmosphere. The individual $COF_{X\text{-}DHTA}$ film-based sensors feature in response rate and response equilibrium time. Therefore, to facilitate the observation and comparison of the stable response values of all the COF films under different humidity conditions, the exposure time was normalized to 100 s (Fig. 4a–f). The recovery times are defined as the time taken by the sensor to achieve 90% of the total current change $|I_{humidity}–I_{dry}|$ in the case of desorption.

Gas sensors based on COF films were assembled for human respiratory monitoring, where human respiration and other related tests are mostly performed as a cycle of every minute/second. In the cycling stability test, the response time was defined as the one-cycle exposure time of 60 s, and the recovery time definition was not changed (Fig. 4g–i). Also, the recovery time is defined as the average of 12-cycles of obtained recovery time.

First, according to the above test procedure, several IDEs-COF film-based sensors were tested for the *I-V* curve in the dynamic test chamber of dry compressed air. The IDE-X-$COF_{TAPB\text{-}DHTA}$-based sensors follow the same test method to obtain the *I-V* curves.

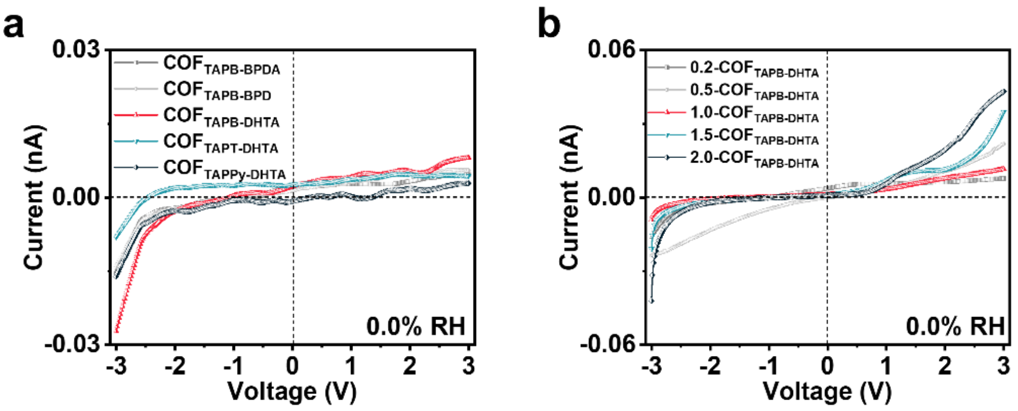

Fig. S7 a *I-V* curves of COF$_{TAPB-BPDA}$, COF$_{TAPB-PDA}$, COF$_{TAPB-DHTA}$, COF$_{TAPT-DHTA}$, and COF$_{TAPPy-DHTA}$ film under ± 3 V bias in dry compressed air atmosphere; **b** *I-V* curves of 0.2-COF$_{TAPB-DHTA}$, 0.5-COF$_{TAPB-DHTA}$, 1.0-COF$_{TAPB-DHTA}$, 1.5-COF$_{TAPB-DHTA}$ and 2.0-COF$_{TAPB-DHTA}$ powder under ± 3 V bias in dry compressed air atmosphere

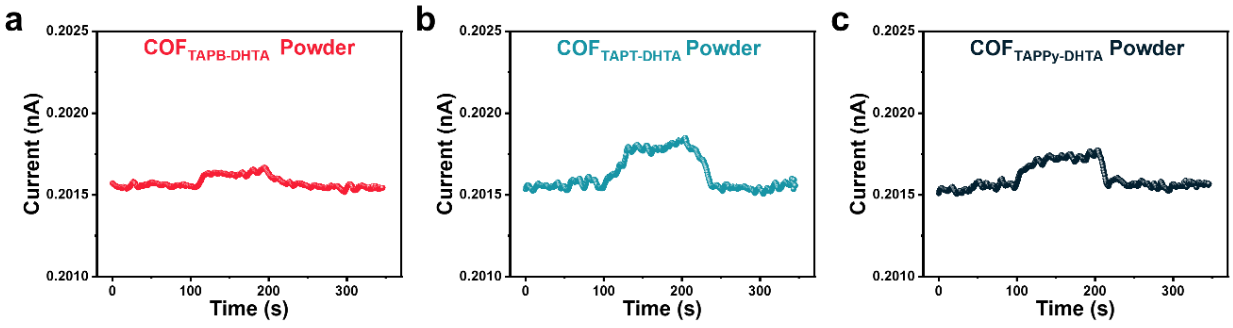

Fig. S8 a Dynamic response characteristic curves of IDEs- COF$_{TAPB-DHTA}$, **b** COF$_{TAPT-DHTA}$ and **c** COF$_{TAPPy-DHTA}$ powder-based humidity sensors from dry air 0.0 to 74.9% RH

To investigate whether there is an interaction between COF thickness and sensing performance. First, the thicknesses of the X-COF$_{TAPB-DHTA}$ series films were determined using an ultra-depth of field 3D microscope (Fig. S9). The thicknesses of the 0.2-COF$_{TAPB-DHTA}$, 0.5-COF$_{TAPB-DHTA}$, 1.0-COF$_{TAPB-DHTA}$, 1.5-COF$_{TAPB-DHTA}$, and 2.0-COF$_{TAPB-DHTA}$ film are 26.92, 65.56, 76.74, 108.9 and 160 μm, respectively. This series of film thickness growth trends are consistent with the pre-designed ones.

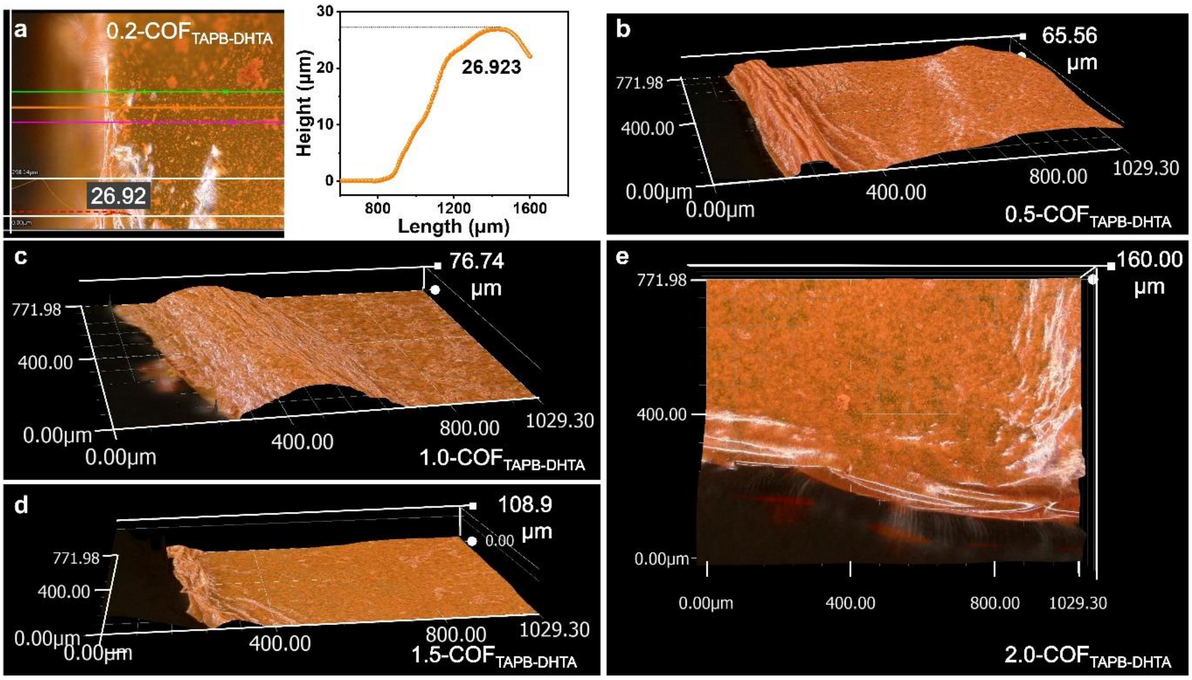

Fig. S9 **a** The surface morphology of 0.2-$COF_{TAPB\text{-}DHTA}$, **b** 0.5-$COF_{TAPB\text{-}DHTA}$, **c** 1.0-$COF_{TAPB\text{-}DHTA}$, **d** 1.5-$COF_{TAPB\text{-}DHTA}$, and **e** 2.0-$COF_{TAPB\text{-}DHTA}$ film in 3D view

Under the same test conditions (0.0−61.2% RH), the humidity sensing response value increases and then decreases as the X-$COF_{TAPB\text{-}DHTA}$ film thickness increases. Among them, 1.5-$COF_{TAPB\text{-}DHTA}$ film exhibited the highest response value of 73.555. This may be related to the degree of crystallization of COF and residual ligand monomers on the surface, which affects the sensing performance of the COF films. Therefore, the 1.5-$COF_{TAPB\text{-}DHTA}$ film is used later as an application demonstration. If there is no special explanation, all 1.5-$COF_{TAPB\text{-}DHTA}$ will be abbreviated as $COF_{TAPB\text{-}DHTA}$ film.

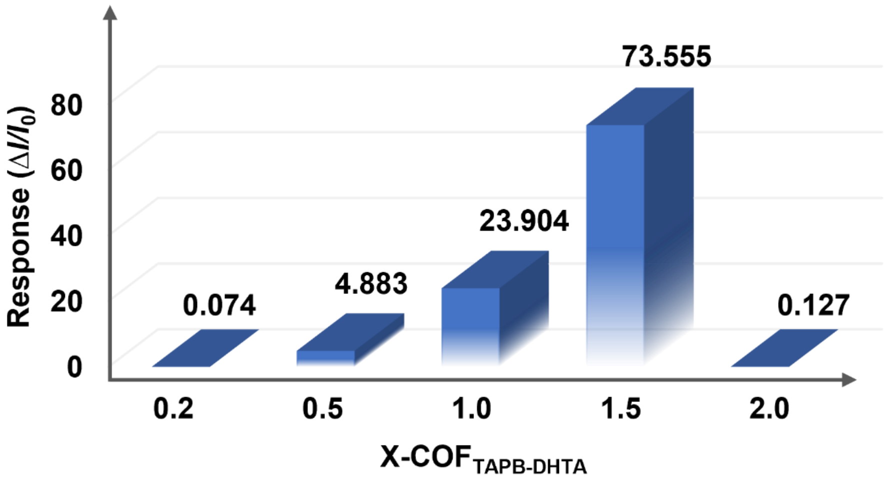

Fig. S10 Dynamic response characteristic curves of IDEs-0.2-$COF_{TAPB\text{-}DHTA}$, 0.5-$COF_{TAPB\text{-}DHTA}$, 1.0-$COF_{TAPB\text{-}DHTA}$, 1.5-$COF_{TAPB\text{-}DHTA}$ and 2.0-$COF_{TAPB\text{-}DHTA}$ film-based humidity sensors from dry air 0.0 to 61.2% RH

For the cyclic stability characteristics of COF film-based humidity sensing, a response was performed every 60 s for a total of 12 cycles. The relative humidity was stabilized at 76.2% ± 3% by adjusting the ratio of carrier gas and background gas. In particular, the autonomous recovery was performed only by placing the COF film-based sensors under ambient conditions without any background external force.

The selectivity experiments of the sensor were performed by rapid evaporation of organic vapors through a high-temperature heating plate and diffusion into the chamber, extrapolated to about 100 ppm for each vapor based on the size of the chamber and the amount of liquid added for each vapor.

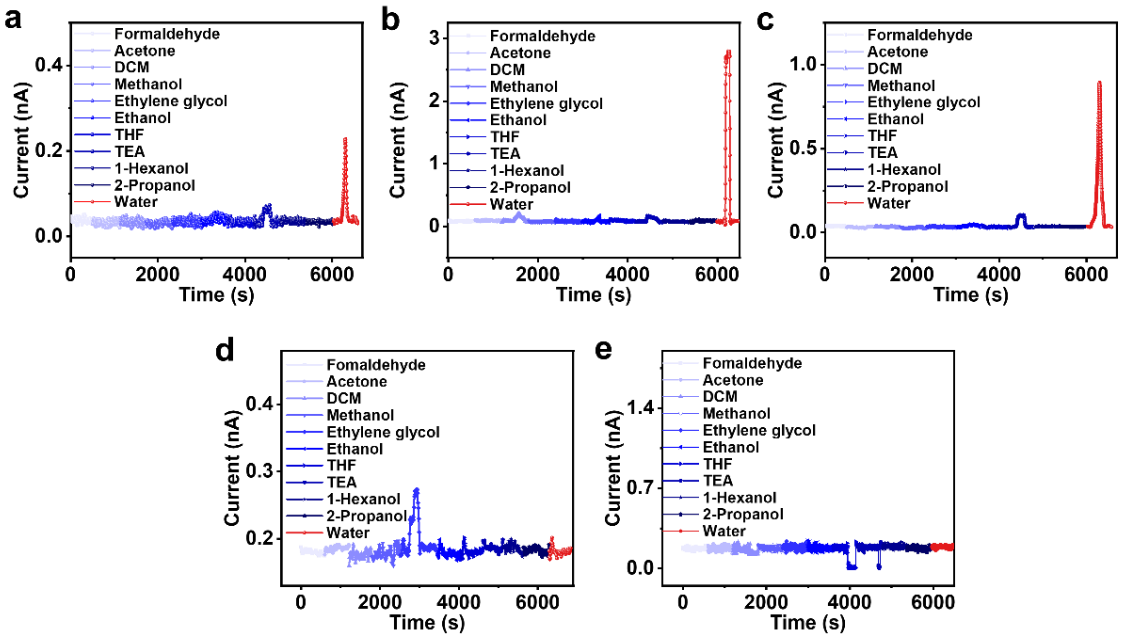

Fig. S11 a Real-time current curves of $COF_{TAPB\text{-}DHTA}$, **b** $COF_{TAPT\text{-}DHTA}$, **c** $COF_{TAPPy\text{-}DHTA}$, **d** $COF_{TAPB\text{-}BPDA}$, and **e** $COF_{TAPB\text{-}PDA}$ film to different gas vapors (the RH range of the test environment is 15–30%)

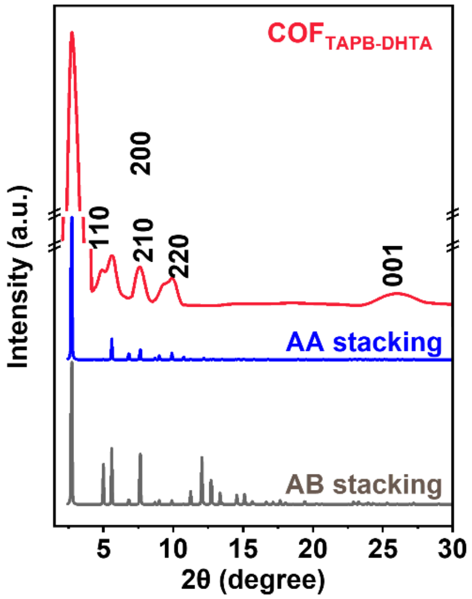

Fig. S12 PXRD patterns of $COF_{TAPB\text{-}DHTA}$ film based on the experimental result marked in red, and simulated AB and AA stacking in blue and grey, respectively

The characteristic diffraction peaks of the (100) face, displayed by the $COF_{TAPB-BPDA}$ and $COF_{TAPB-PDA}$ film correspond to 2.92° and 2.90°, respectively. It was proved that the comparative COF films were successfully prepared, and the diffraction characteristic peaks were consistent with the literature [S1]. The PXRD of the two contrasting materials, $COF_{TAPB-BPDA}$ and $COF_{TAPB-PDA}$ film, both showed excellent crystallinity. The above results prove that the crystallization degree of the comparative COF films is still well, to exclude the factor on the crystallization degree of the COF films.

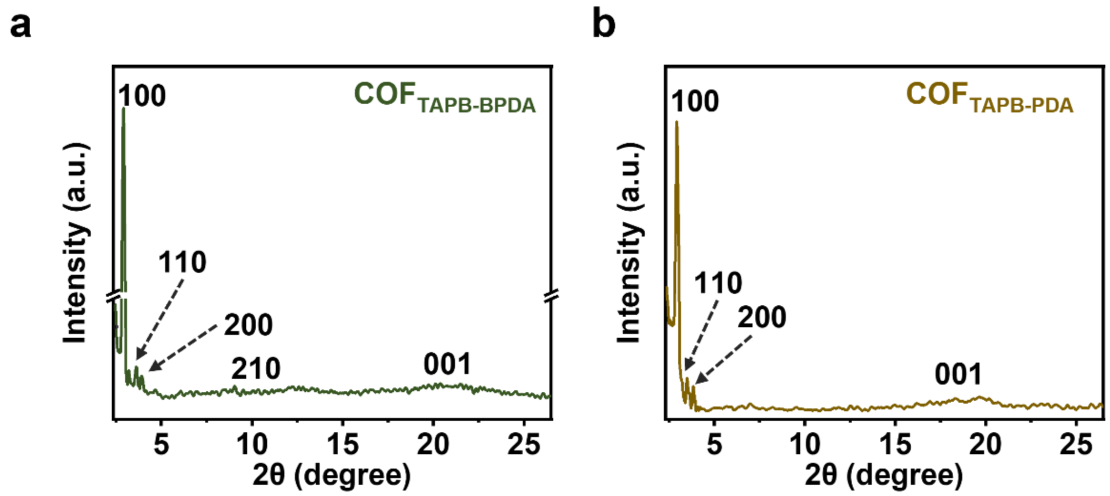

Fig. S13 a PXRD patterns of $COF_{TAPB-BPDA}$ and **b** $COF_{TAPB-PDA}$ film

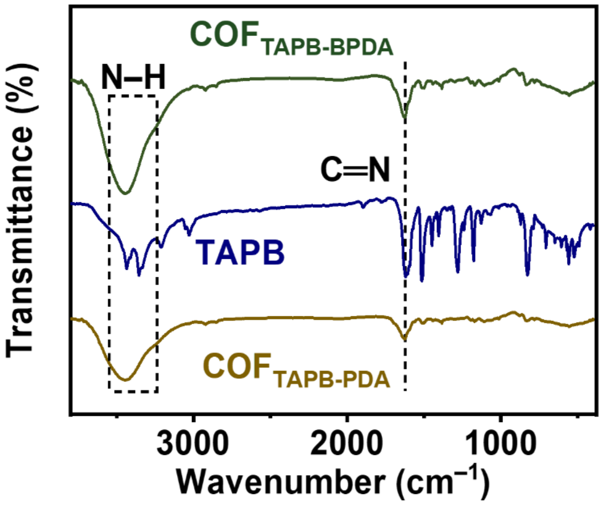

Fig. S14 FT-IR spectra of $COF_{TAPB-BPDA}$ and $COF_{TAPB-PDA}$ film

To evaluate the chemical components of the prepared COF_{X-DHTA} films, XPS analysis was performed. The full XPS spectra of the COF_{X-DHTA} films all indicate the existence of only three elements, C, N, and O (Fig. S15a, e, and i), where the peak positions of each element are very similar. As shown in Fig. S15b, the core-level C 1s XPS spectrum of $COF_{TAPB-DHTA}$ could be separated into three main parts; i.e., peaks located at 284.4, 285.9, and 288.1 eV. These peaks are ascribed to C=C, C=N−C, and N−C=O, respectively, indicating that $COF_{TAPB-DHTA}$ possesses a π-conjugated structure [S2]. The N 1s core-level XPS spectrum of $COF_{TAPB-DHTA}$ (Fig. S15c) could be fitted into three main parts, i.e., the peaks at 398.9 and 400.5 eV, along with another weak peak at 404.4 eV, corresponding to atomic imine N, pyridinic N, and pyridinic $-(N^{+}-O^{-})$, respectively [S3]. In the case of $COF_{TAPPy-DHTA}$ film (Fig. S15k), only one

peak at 399.4 (pyrrolic N) is observed. Here, the pyrrolic N contributes to the electron density of the π-conjugated system with a pair of p-electrons in the COF layers. The O 1s core-level XPS spectrum of $COF_{TAPB-DHTA}$ (Fig. S15d) shows two peaks at 529.5 and 531.1 eV, which are due to C=O and C−O, respectively. The result suggests that oxygen is introduced by DHTA, thus confirming the chemical reaction between TAPB and DHTA.

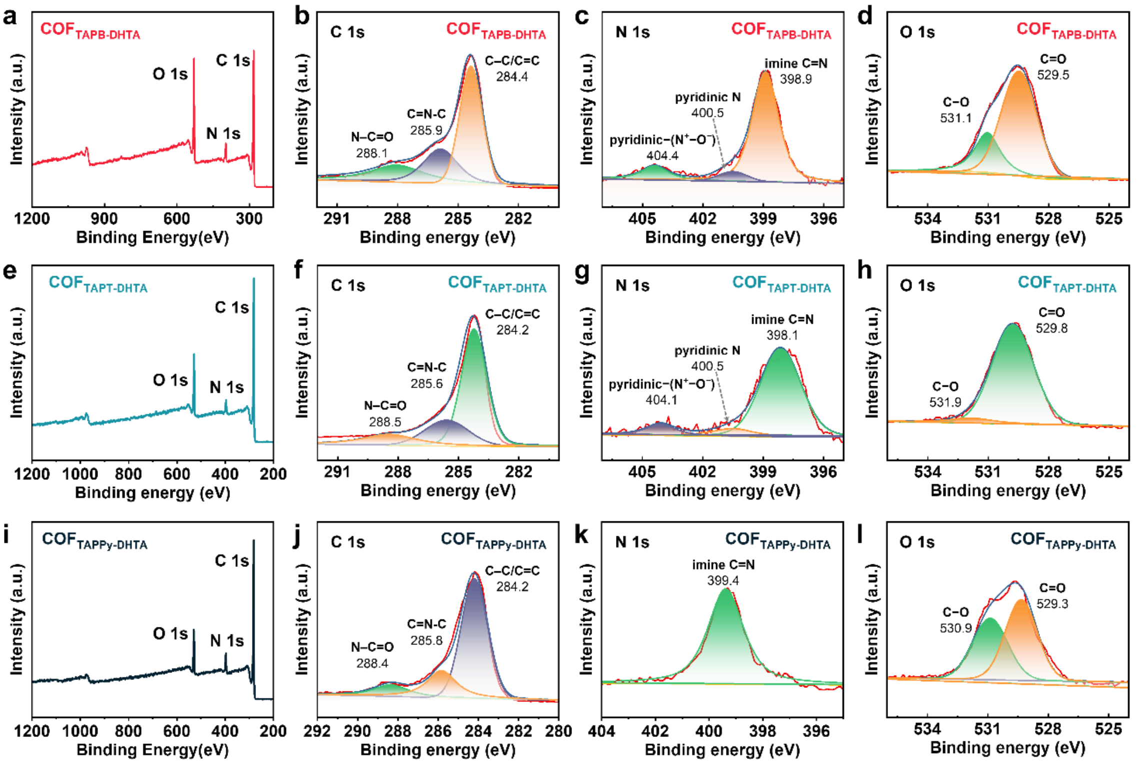

Fig. S15 a−d XPS spectra of $COF_{TAPB-DHTA}$, **e−h** $COF_{TAPT-DHTA}$, and **i−l** $COF_{TAPPy-DHTA}$ film

Under the same preparation conditions as $COF_{TAPB-DHTA}$, the AFM topography of $COF_{TAPT-DHTA}$ was observed as particles and a nanosheet with a thickness of 3.996 nm. However, in the range of 4 μm × 4 μm in the $COF_{TAPPy-DHTA}$ AFM image, only the bulk of nanosheet stacks can be obtained. The three-layer nanosheets stacked within the range of the orange line in Fig. S16c with heights of 3.448, 3.332 and 2.855 nm from top to bottom, respectively. Compared with $COF_{TAPB-DHTA}$ and $COF_{TAPPy-DHTA}$ film, the 3D depiction of the topography is more vivid, and the dispersion and nanosheet uniformity of the former is superior.

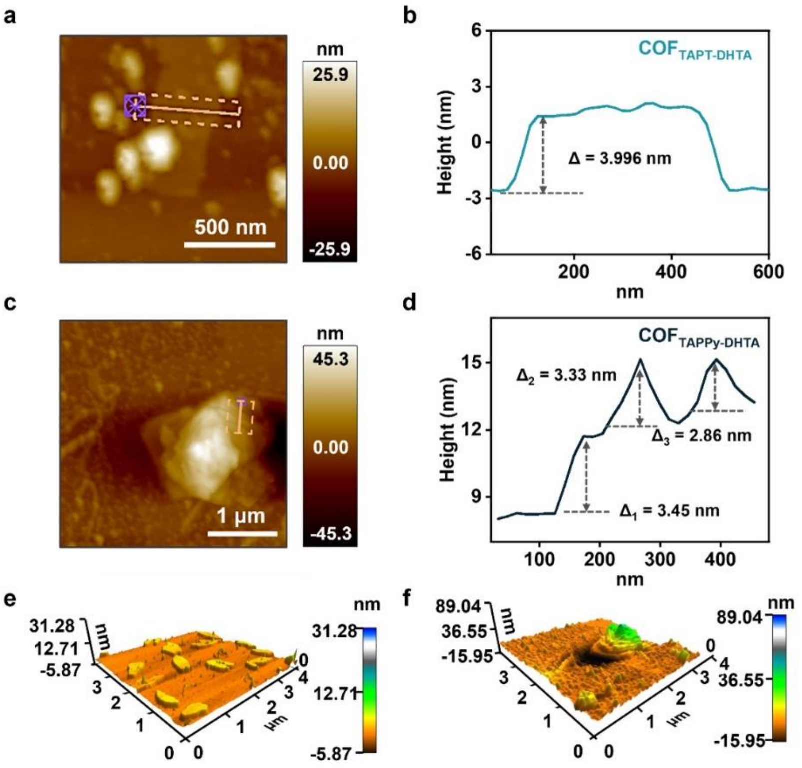

Fig. S16 AFM topography image of **a** $COF_{TAPT\text{-}DHTA}$. **b** The corresponding height curve of $COF_{TAPT\text{-}DHTA}$. **c** AFM topography image of $COF_{TAPPy\text{-}DHTA}$ film. **d** The corresponding height curve of $COF_{TAPPy\text{-}DHTA}$ film. **e** 3D depiction of the $COF_{TAPB\text{-}DHTA}$ and **f** $COF_{TAPPy\text{-}DHTA}$ film topography (scale bar is 4 μm)

The $COF_{TAPB\text{-}BPDA}$ and $COF_{TAPB\text{-}PDA}$ films exhibited porous morphologies, and the powders also exhibited uniform sphere arrangement. However, the regular microscopic morphology of these COFs does not introduce humidity-sensing properties. Therefore, it is the pre-design of the molecular level of COF, through the selection of monomers and functional groups, that plays a dominant role in the sensing performance.

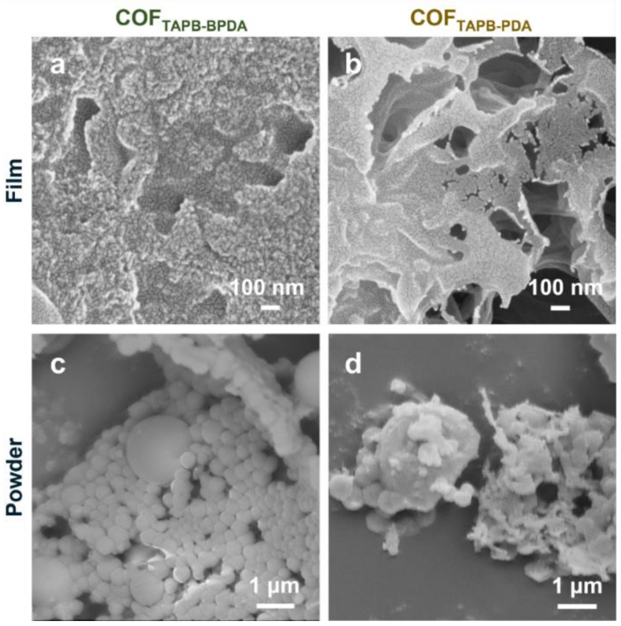

Fig. S17 a SEM images of $COF_{TAPB\text{-}BPDA}$ film, **b** $COF_{TAPB\text{-}PDA}$ film, **c** $COF_{TAPB\text{-}BPDA}$, and **d** $COF_{TAPB\text{-}PDA}$ powders

The flat cross-sectional structure of COFX-DHTA films can be observed by SEM images, which can be tightly bonded to the IDEs surface, and provide the basis for stable output of sensing signal. Since the COF films are formed by the continuous stacked growth of nanospheres, several nanoparticles are shown in the cross-sectional view.

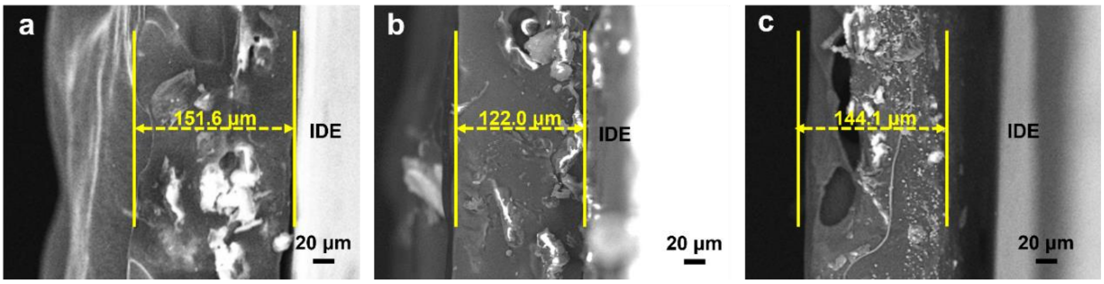

Fig. S18 a SEM images of the cross-sectional view of $COF_{TAPB-DHTA}$, **b** $COF_{TAPT-DHTA}$, and **c** $COF_{TAPPy-DHTA}$

IFFT analysis of the red rectangle in Fig. 3h from the $COF_{TAPB-DHTA}$ film was performed by Digital Micrograph software. It is demonstrated that the crystal particles were cross-stacked together to form an obvious network diagram. After measurement, the distance of the five lattice fringes is 1.492 nm, and the calculated average lattice spacing is 2.98 nm, which is almost consistent with the (100) crystal plane corresponding to PXRD.

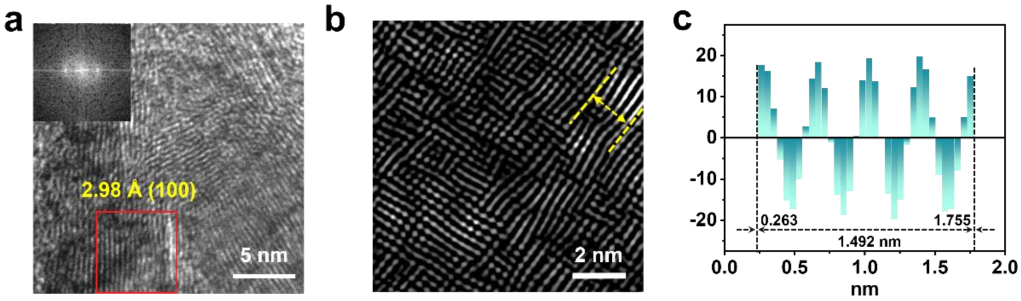

Fig. S19 a HR-TEM image of $COF_{TAPB-DHTA}$ (insert: simulated electron diffraction pattern through the fast Fourier transform from the red rectangle on the $COF_{TAPB-DHTA}$ film). **b** IFFT and **c** lattice profile analyses by the Digital Micrograph of $COF_{TAPB-DHTA}$ film

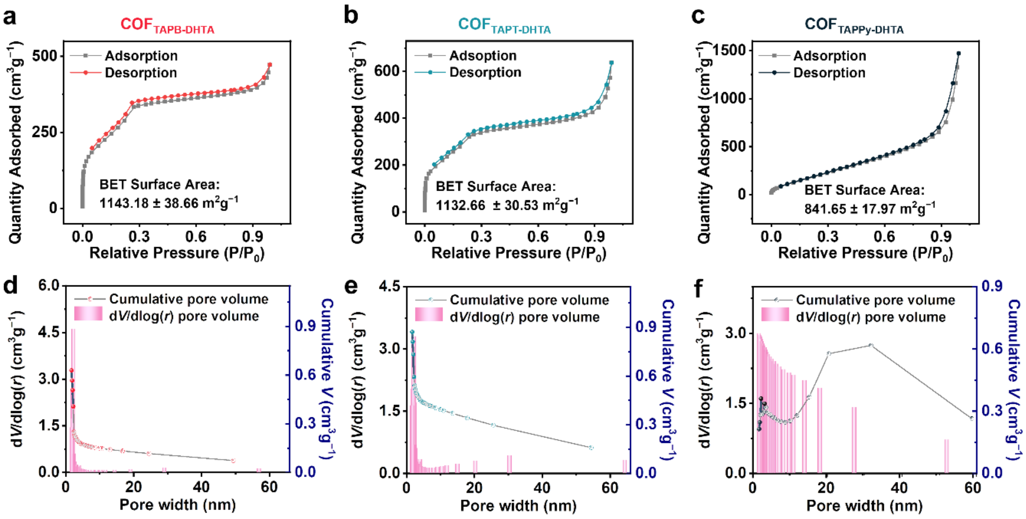

Fig. S20 a Nitrogen sorption isotherm curves of $COF_{TAPB-DHTA}$, **b** $COF_{TAPT-DHTA}$, and **c** $COF_{TAPPy-DHTA}$ film. The pore size distribution of **d** $COF_{TAPB-DHTA}$, **e** $COF_{TAPT-DHTA}$, and **f** $COF_{TAPPy-DHTA}$ film

Compared to $COF_{X\text{-}DHTA}$ films, both $COF_{TAPB\text{-}PDA}$ and $COF_{TAPT\text{-}BPDA}$ films have smaller BET results, with relatively dispersed pore size distributions. From the perspective of the topological structure, it shows that the DHTA coordination monomer with the hydroxyl group has the advantage in the interfacial self-assembly at room temperature.

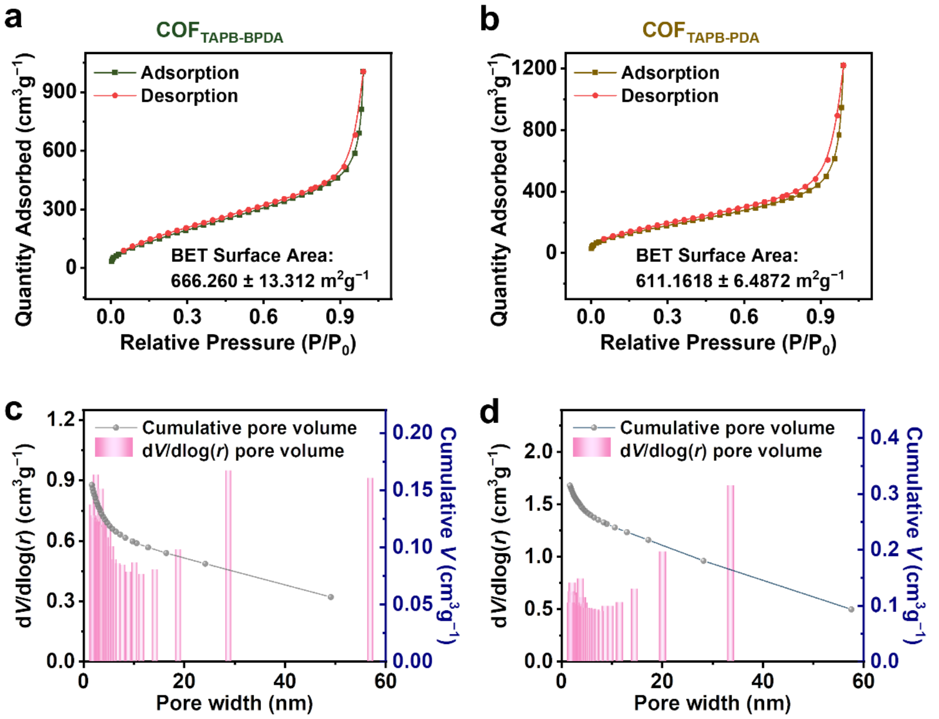

Fig. S21 **a** Nitrogen sorption isotherm curves of $COF_{TAPB\text{-}BPDA}$ and **b** $COF_{TAPB\text{-}PDA}$ film. The pore size distribution of **c** $COF_{TAPB\text{-}BPDA}$ and **d** $COF_{TAPB\text{-}PDA}$ film

The IDE-$COF_{TAPB\text{-}DHTA}$ film-based humidity sensor has proven good structural stability through long-term cycling tests. The COF conjugate stabilized frame advantage allows its sensors a long service life in complex atmospheric environments.

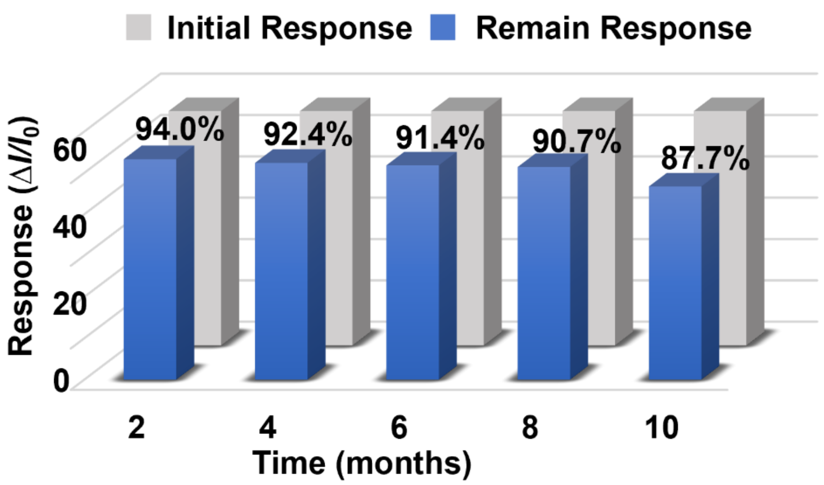

Fig. S22 The long-term response stability of the IDE-$COF_{TAPB\text{-}DHTA}$ film-based humidity sensor

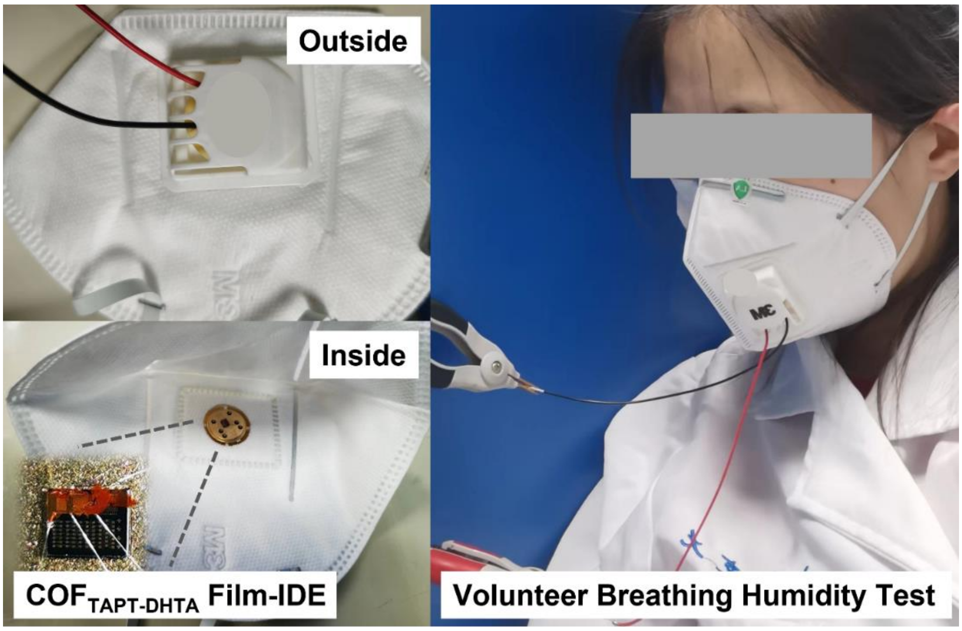

Fig. S23 Optical photographs of the outside and inside of a and wear a mask with a breathing valve embedded in the IDEs-COF$_{TAPB-DHTA}$ film-based humidity sensor to monitor the respiratory humidity of Volunteer-I

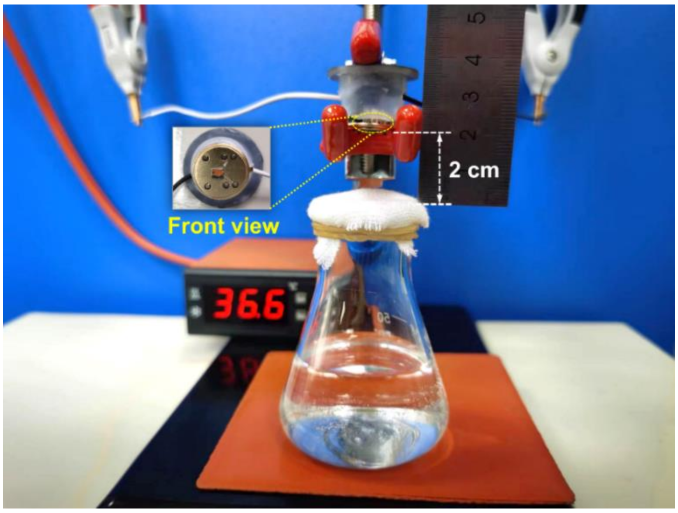

Fig. S24 Photograph of the experimental setup of the IDEs-COF$_{TAPB-DHTA}$ film-based humidity sensors used to simulate the breathability of fabrics at human body temperature

The IDE-COF$_{X-DHTA}$ device was placed in a test chamber of 12 cm × 12 cm × 12 cm = 1728 cm^3 to check the response toward a mixture of gases. Excluding the possibility of organic solutions containing water, all experiments were performed using standard gas concentrations. The concentration of each standard gas is known to be 2000 ppm, and 86.4 mL of H_2S, SO_2, CO, CO_2, and NH_3 (100 ppm of each gas in the chamber) was injected using a syringe and in segments, the chamber was kept sealed after each injection. It is noticed that the COF$_{X-TAPB}$-based sensors produced almost no response within the gas mixture atmosphere. Waiting for the output signal to be stabilized, compressed air carrying a specific humidity (~51.7% RH) was introduced and purged for 60 s per exposure. After three cycles, the IDE-COF$_{X-DHTA}$ device maintained a stable response signal to water vapor within the mixed gas atmosphere. The COF film-based sensors were demonstrated to be selective and ultra-sensitive to water vapor under complex human breathing conditions (Fig. S25).

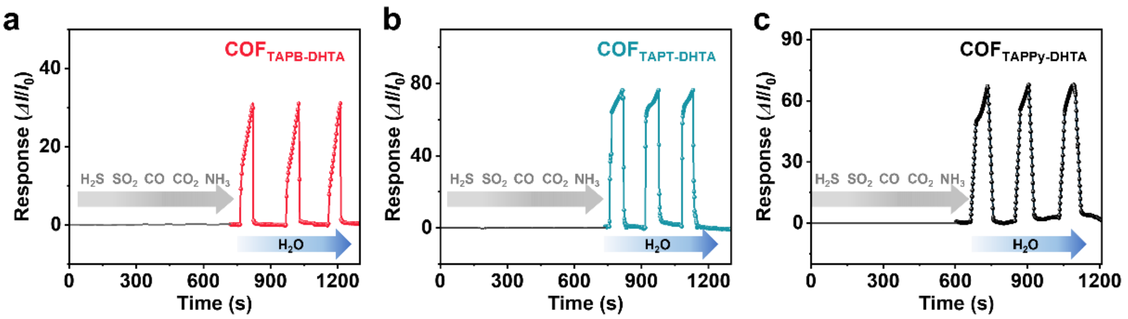

Fig. S25 a Dynamic response curves of the IDE-COF$_{TAPB-DHTA}$, **b** IDE-COF$_{TAPT-DHTA}$, and **c** IDE-COF$_{TAPPy-DHTA}$ devices to 51.7% RH in a chamber containing H_2S, SO_2, CO, CO_2, and NH_3, with a concentration of 100 ppm for each gas

The sensors based on IDE-COF$_{X-DHTA}$ were placed at a vertical position 3 cm away from the light source for real-time measurements at room temperature, where the wavelengths of the light sources were 700, 550, 420, and 365 nm, respectively, with a power of 1.5 W. To confirm the accuracy of the light response, the exposure was repeated three times with each light source for about 60 s each time. In general, the light exposure had almost no effect on the three IDE-COF$_{X-DHTA}$ film-based sensors. Because of the minimal band gap caused by the triazine monomer, the IDE-COF$_{TAPT-DHTA}$-based sensor is more vulnerable to exciton jumps and current fluctuations [S4]. Therefore, its' curve shows slight fluctuations during light exposure. It is worth mentioning that the absorption spectra of several COF films were tested under UV-Vis measurement and they have specific absorption curves, but this does not mean that all materials have photo-responsive properties.

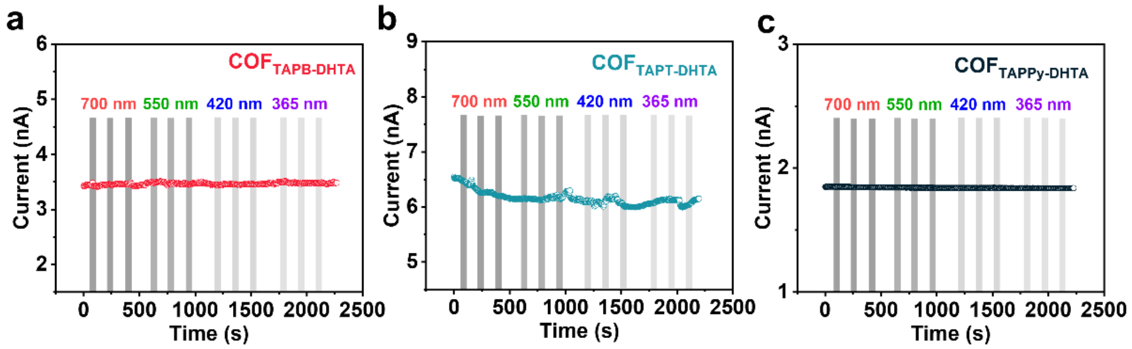

Fig. S26 a Dynamic current curves of the IDE-COF$_{TAPB-DHTA}$, **b** IDE-COF$_{TAPT-DHTA}$, and **c** IDE-COF$_{TAPPy-DHTA}$ devices to the light with 700, 550, 420, and 365 nm (atmospheric condition 64% RH)

The COF$_{X-DHTA}$ film-based devices were tested at ~ 50% RH response curves at 30, 20, and 10 °C ambient temperature controlled by central air conditioning. The high-temperature conditions were tested by connecting the device base with a ceramic heating plate (R = 2 Ω, Vmax = 10 V) at 0.5, 1.5, and 2.0 V corresponding to 95, 130, and 180 °C, respectively. First, the humidity response values and the underlying current curves are almost invariable at 30, 20, and 10 °C in room temperature environments. However, under high-temperature conditions, the resistance of the metal electrodes changes at high temperatures, increasing the resistance of the sensor devices as the temperature rises and a decrease in the output current signal, accompanied by a decrease in response. In general, the ambient temperature is almost impossible to reach such a high temperature, so the COF film humidity sensor has almost no response disturbance in a room temperature environment.

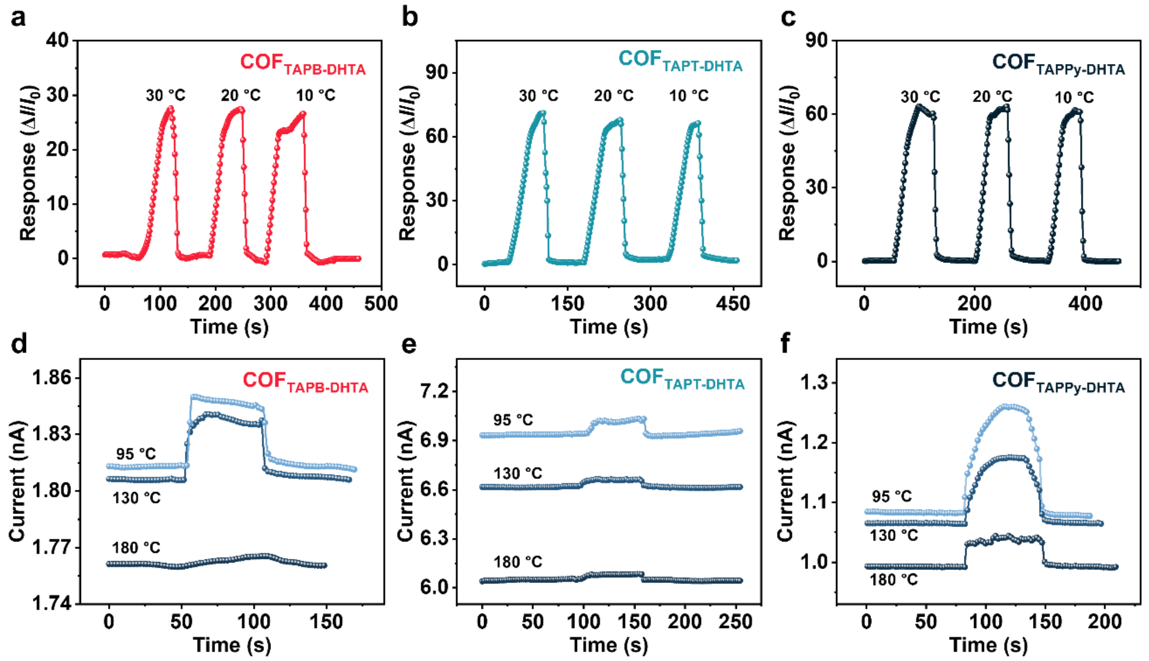

Fig. S27 a Dynamic response curves of the IDE-COF$_{TAPB-DHTA}$, **b** IDE-COF$_{TAPT-DHTA}$, and **c** IDE-COF$_{TAPPy-DHTA}$ devices to 50% RH at 30, 20, and 10 °C. **d−e** IDE-COF$_{X-DHTA}$ devices to 50% RH at 95, 130, and 180 °C

These three COF$_{X-DHTA}$ films exhibited only slight hysteretic response behaviors observed with steep variations over narrow ranges of RH, indicating the good reversibility of moisture sorption. The dynamic properties curves of COF$_{X-DHTA}$ films demonstrate their potential in humidity-sensing applications.

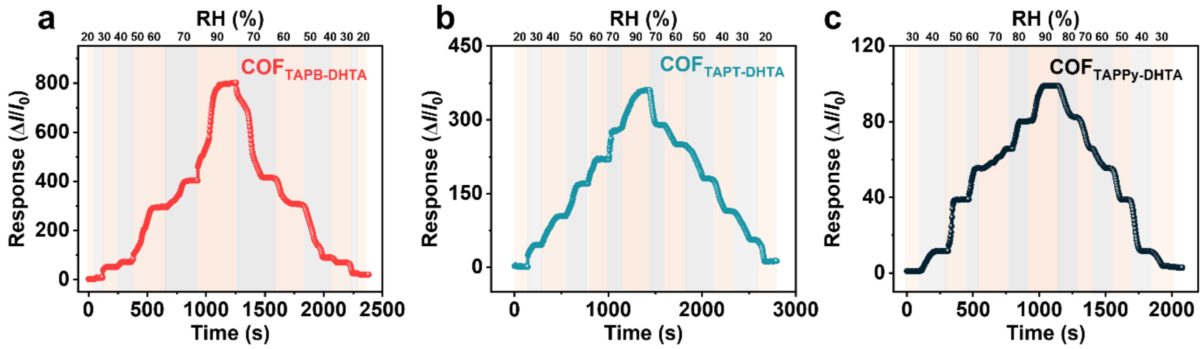

Fig. S28 a Dynamic water vapor response-recovery kinetics of COF$_{TAPB-DHTA}$, **b** COF$_{TAPT-DHTA}$, and **c** COF$_{TAPPy-DHTA}$ film

Hysteresis loss is an important parameter for evaluating sensor performance and sensor stability. Herein, the hysteresis loss is described as the shift in response value as the sensor is subjected to a change in RH level from a lower RH level to a higher RH level (absorption process) and from a higher RH level to a lower RH level (desorption process). As shown in Fig. S29, the COF$_{X-DHTA}$ film-based humidity sensors show low hysteresis losses. Among them, COF$_{TAPPy-DHTA}$ exhibits extremely high stability due to its topology which is smaller and has lower energy than the hexagonal COF$_{TAPB-DHTA}$ and COF$_{TAPT-DHTA}$.

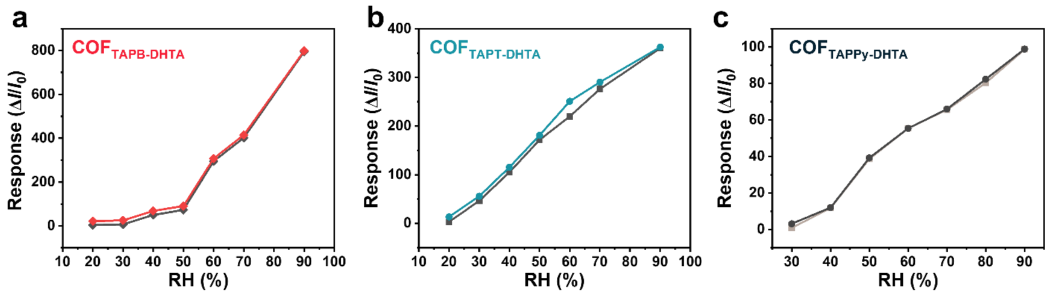

Fig. S29 a Hysteresis response of $COF_{TAPB-DHTA}$, **b** $COF_{TAPT-DHTA}$, and **c** $COF_{TAPPy-DHTA}$ film-based current humidity sensors are operated at 25 ± 1 °C

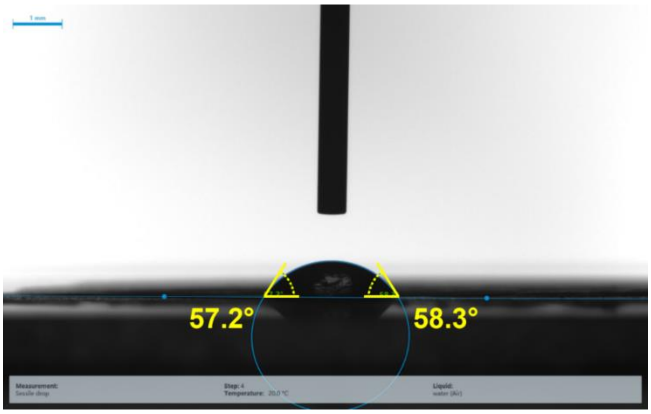

Fig. S30 Contact angle photograph of the $COF_{TAPB-DHTA}$ film

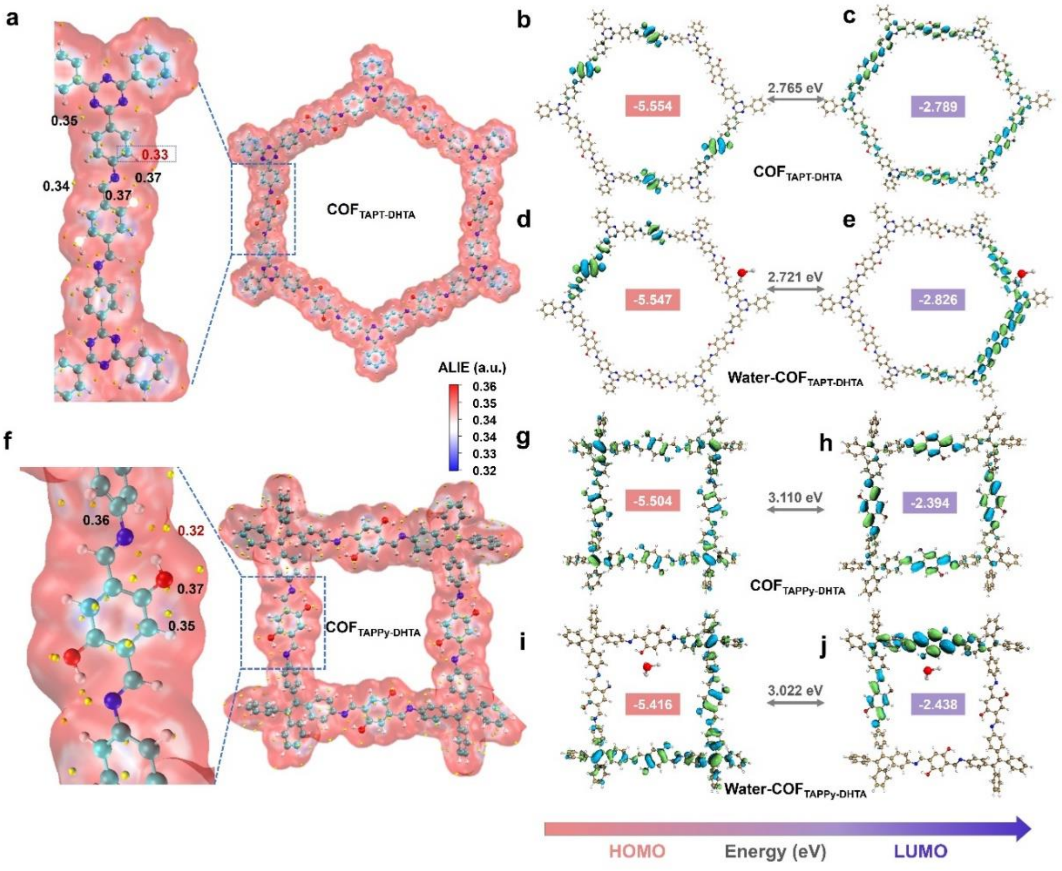

Fig. S31 a ALIE distribution on the surface of $COF_{TAPT-DHTA}$ film. **b** HOMO and **c** LUMO of $COF_{TAPT-DHTA}$ film. **d** W-HOMO and **e** W-LUMO of Water-$COF_{TAPT-DHTA}$ is formed by

absorbing water molecules of $COF_{TAPT-DHTA}$ film. **f** ALIE distribution on the surface of $COF_{TAPPy-DHTA}$ film. **g** HOMO and **h** LUMO of $COF_{TAPPy-DHTA}$ film. **i** W-HOMO and **j** W-LUMO of Water-$COF_{TAPPy-DHTA}$ formed by absorbing a water molecule of $COF_{TAPPy-DHTA}$ film

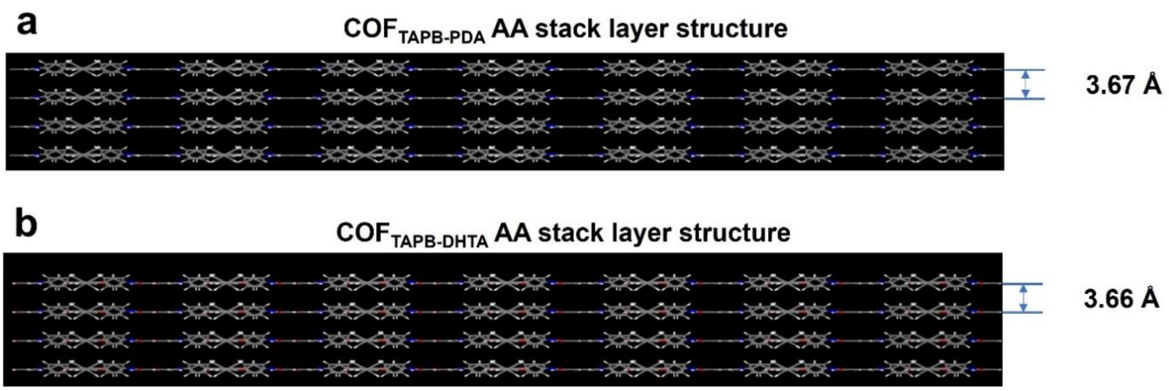

Fig. S32 a The AA stack layer structure of $COF_{TAPB-PDA}$ and **b** $COF_{TAPB-DHTA}$

The DRS absorption spectra (Fig. S33) reveal that the COF films exhibited obvious optical absorption at around 695 nm in the region of 200–1200 nm. The band gap energy could be computed from the formula $(\alpha h\nu)^2 \propto h\nu - E_g$, where α, h, ν, and E_g were the absorption coefficient, Planck's constant, light frequency, and band gap energy, respectively. By ranking the band gaps of several COF films in the dry condition, the results are as follows: $COF_{TAPT-DHTA}$ (1.50 eV) < $COF_{TAPB-BPDA}$ (1.53 eV) < $COF_{TAPB-PDA}$ (1.54 eV) < $COF_{TAPPy-DHTA}$ (1.55 eV) < $COF_{TAPB-DHTA}$ (1.56 eV). Among them, $COF_{TAPT-DHTA}$ with the triazine structure exhibits the smallest band gap value, which shows consistent with UPS. Sensitivity, the difference of resistance generated by the adsorption of the target analyte, is the function of adsorption energy and mobility. It is necessary to secure high carrier mobility with a narrowed band gap and water molecules' adsorption energy. Therefore, preparing COF films with high water molecules' adsorption energy should have an appropriate band gap. Consequently, although the band gap of $COF_{TAPB-DHTA}$ is the largest in the ordering, it can have the excellent humidity-sensitive capability. In addition, the band gaps of $COF_{TAPB-BPDA}$ and $COF_{TAPB-PDA}$ are smaller, but they cannot provide hydroxyl active functional groups due to the lack of DHTA monomers in the framework. It shows that the topological structure and functional groups of COF films play a key role in humidity sensing performance.

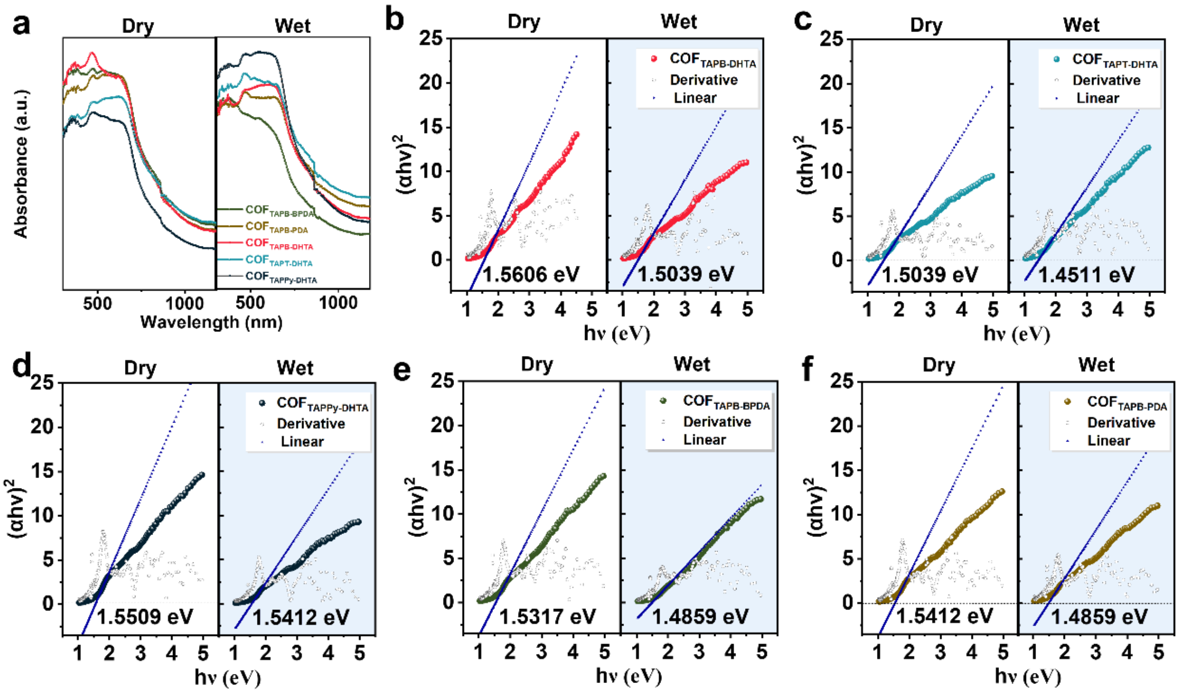

Fig. S33 a UV–vis DRS of COF films at dry and wet conditions. **b** The corresponding plots of the $(ahv)^2$ versus photon energy (hv) for $COF_{TAPB\text{-}DHTA}$, **c** $COF_{TAPT\text{-}DHTA}$, **d** $COF_{TAPPy\text{-}DHTA}$, **e** $COF_{TAPB\text{-}BPDA}$, and **f** $COF_{TAPB\text{-}PDA}$

The energy level of the valence band maximum (E_{VB}) and ultraviolet photoelectron spectroscopy (UPS) of the three COF films were also performed, and the results are displayed in Fig. S34. Employing subtracting the UPS width from excitation energy, the E_{VB} vs. vacuum energy level was calculated to be 3.04, 2.98, and 3.07 eV for $COF_{TAPB\text{-}DHTA}$, $COF_{TAPT\text{-}DHTA}$, and $COF_{TAPPy\text{-}DHTA}$ film, respectively. The corresponding E_{VB} positions of $COF_{TAPB\text{-}DHTA}$, $COF_{TAPT\text{-}DHTA}$, and $COF_{TAPPy\text{-}DHTA}$ film were 1.26, 1.21, and 1.26 eV, respectively.

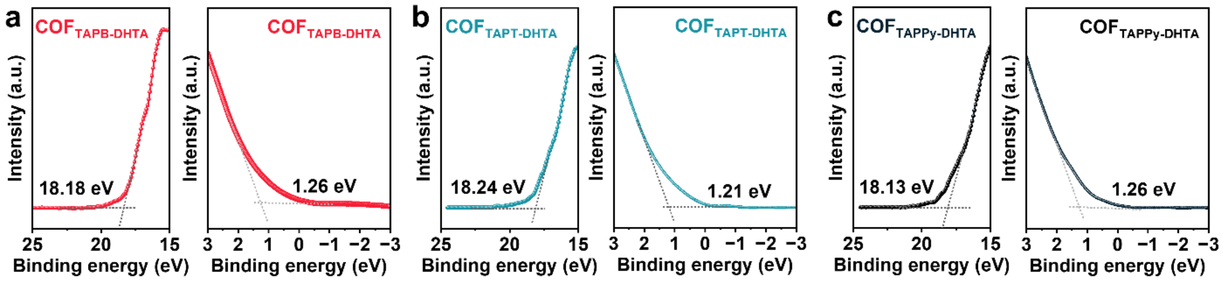

Fig. S34 a UPS spectra of $COF_{TAPB\text{-}DHTA}$, **b** $COF_{TAPT\text{-}DHTA}$, and **c** $COF_{TAPPy\text{-}DHTA}$ film. **d** The estimated band structure of $COF_{TAPB\text{-}DHTA}$, $COF_{TAPT\text{-}DHTA}$, and $COF_{TAPPy\text{-}DHTA}$ film

The band structure alignment was summarized in Fig. S35. And the conduct band minimum (CBM) position of $COF_{X\text{-}DHTA}$ film was similarly located around −0.3 eV. $COF_{TAPT\text{-}DHTA}$ possessed the smallest band bap, which is consistent with the trend of the DFT simulations.

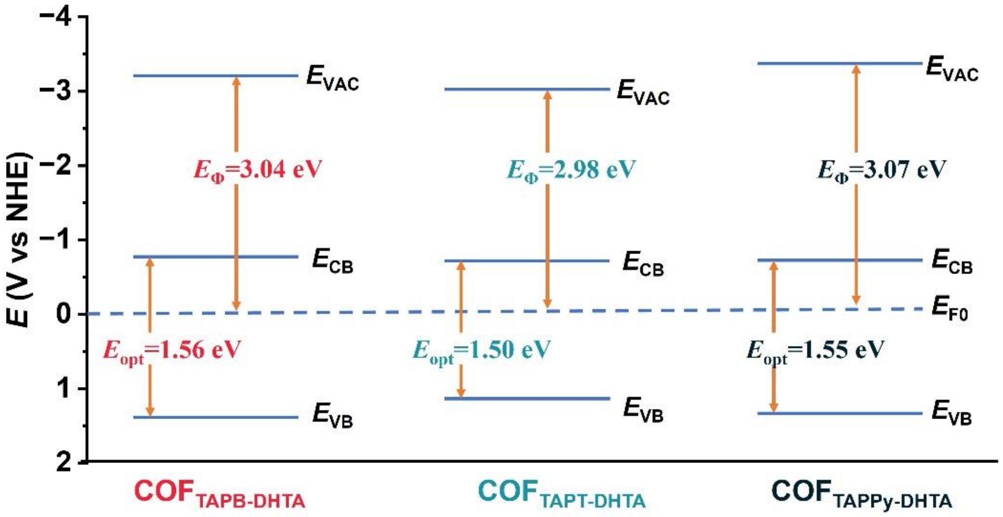

Fig. S35 The band structure of $COF_{TAPB\text{-}DHTA}$, $COF_{TAPT\text{-}DHTA}$, and $COF_{TAPPy\text{-}DHTA}$ film according to UPS and UV-Vis DRS spectra

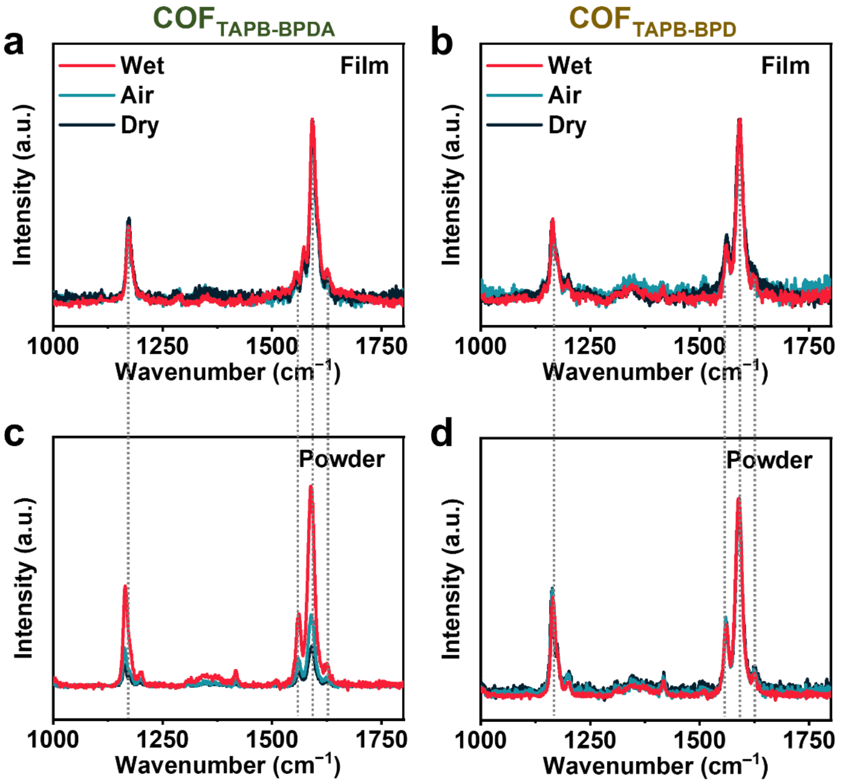

Fig. S36 **a** *In situ* Raman spectra of $COF_{TAPB\text{-}BPDA}$ and **b** $COF_{TAPB\text{-}PDA}$ film. *In situ* Raman spectra of **c** $COF_{TAPB\text{-}BPDA}$ and **d** $COF_{TAPB\text{-}PDA}$ powder

Based on the above theoretical and experimental analysis, the humidity sensing mechanism of $COF_{X\text{-}DHTA}$ films is shown in Fig. S37 and summarized as follows. The excellent response of $COF_{X\text{-}DHTA}$ film to humidity is mainly due to the existence of imine bonds in the skeleton, and the N atoms as the active sites combining with water molecules to form iminol/*cis*-ketoenamine isomer. The E_g difference before and after the adsorption of water molecules is only -0.19 eV by DFT calculation, leading to a completely reversible response at room temperature. With the high specific surface area and continuous pores distribution of $COF_{X\text{-}DHTA}$ films, more N-atom active sites are exposed to interact with water molecules. At the same time, the hydrophilic surface and abundant hydroxyl groups help to capture water

molecules. Under low RH conditions, the empty orbitals of N atoms interact with lone electron pairs of oxygen atoms in water molecules. A small number of discontinuous water molecules form a low percentage of tautomerism on the surface of $COF_{X\text{-}DHTA}$ films. However, at high RH, multilayer adsorption is obtained to form the array of water molecules in the porous channels of $COF_{X\text{-}DHTA}$ films, which facilitates the charge transport process through the Grotthuss chain reaction ($H_2O + H_3O^+ \rightarrow H_3O^+ + H_2O$). A high proportion of the $COF_{X\text{-}DHTA}$ skeleton is converted to the iminol/*cis*-ketoenamine isomer, resulting in a dramatic increase in the conductivity of the sensors. In addition, the electric field ionizes water molecules to generate hydronium ions (H_3O^+) as charge carriers, and the transition of protons ($H_3O^+ \rightarrow H_2O + H^+$) between adjacent water molecules after hydration is also beneficial for humidity sensing. Finally, the Raman spectra of $COF_{TAPB\text{-}DHTA}$ film elucidate that water molecules can induce the dual-site active adsorption of N atoms under high RH conditions, the (C=N) and (C−N) stretching vibrations with ultra-high sensitivity.

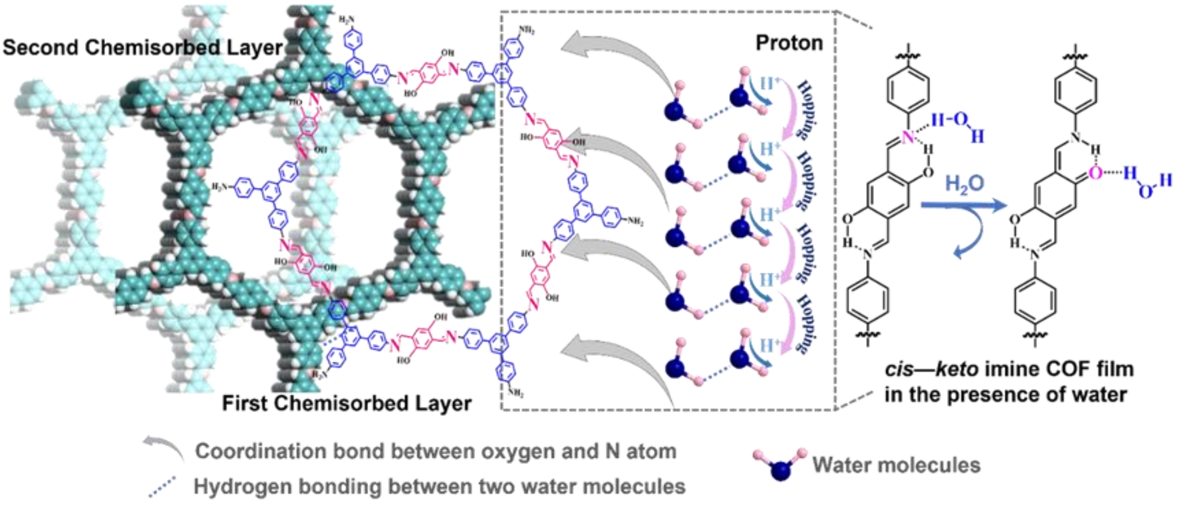

Fig. S37 Mechanism of *cis-Keto* imine tautomerism generated by $COF_{X\text{-}DHTA}$ films interaction with water molecules for humidity sensing

Table S1 A comparison of humidity sensing performance of $COF_{TAPB\text{-}DHTA}$ film with previously reported works

**Active materials**		**Sensor type**	**Device type structure**	**Detection range (%)**	**Detection limit (%)**	t_{res}/t_{rec} **(s)**	**Refs.**
**Carbon material**	biochar	impedance	powder IDE	0–90	–	≈270/≈60	[S5]
	GO	frequency	QCM	10–90	>10	–	[S6]
	GO	capacitance	layers-IDE	10–90	>10	15.8/–	[S7]
	CNF/CNT	resistance	paper-based	11–95	>11	321/435	[S8]
**Conducting polymer**	PVA/KOH	resistance	flexible ionic membrane	10.89–81.75	>11	0.4/2.6	[S9]
	PAM/CA	resistance	hydrogels	4–90	4	0.27/0.3	[S10]
**MOFs**	MIL-96(Al)	capacitance	powder IDE	0–90	3.7	>600/>1000	[S11]

	MOF-76(Gd)	resistance	powder IDE	11–98	>11	11/2	[S12]
**TMDs**	monolayer MoS_2 film	resistance	FETs array	0–35	0	≈10/≈60	[S13]
	WS_2/PDMS	resistance	film IDE	20–90	>20	5/6	[S14]
**MXenes**	MXenes	frequency	powder IDE	13–97	>13	1.11/0.65	[S15]
	Ti_3C_2 MXene film	frequency	QCM	0.1–95	0.1	–	[S16]
**COFs**	TpPa-SO_3Na	impedance	powder IDE	11–95	11	6/69	[S17]
	COF-TXDBA	impedance	powder IDE	11–98	>11	27/42	[S18]
	Py-TT COF	absorbance	films on fused silica	–	–	0.2/–	[S19]
	COF/aramid fabric	color-changes	fibers	20–100	>20	–	[S20]
**Commercial**	GO	capacitive	200 mm CMOS	≈22.5–85	>20	–/≈8	[S21]
	SHT25 (polyimide)	capacitance	IDT	16–85	>16	~1500/40	[S22]
	OMEGA-RH-20C	resistance	–	10–95	>10	–	[S23]
**This work**	COF$_{\text{TAPB-DHTA}}$ Film	resistance	film IDE & Macro film	13–98	>7	0.4/1	–

Table S2 Molecular orbital value of COF$_{\text{TAPB-PDA}}$, Water-COF$_{\text{TAPB-PDA}}$, COF$_{\text{TAPB-DHTA}}$, Water-COF$_{\text{TAPB-DHTA}}$, COF$_{\text{TAPT-DHTA}}$, Water-COF$_{\text{TAPT-DHTA}}$, COF$_{\text{TAPPy-DHTA}}$, and Water-COF$_{\text{TAPPy-DHTA}}$

Samples	Mode	HOMO (eV)	LUMO (eV)	E_g (eV)	ΔE (eV)
**COF$_{\text{TAPB-PDA}}$**	eclipsed AA	-5.87	-2.60	3.27	-0.13
**Water-COF$_{\text{TAPB-PDA}}$**		-5.82	-2.68	3.14	
**COF$_{\text{TAPB-DHTA}}$**	eclipsed AA	-5.71	-2.48	3.23	**-0.19**
**Water-COF$_{\text{TAPB-DHTA}}$**		-5.69	-2.65	3.04	
**COF$_{\text{TAPT-DHTA}}$**	eclipsed AA	-5.554	-2.789	2.765	-0.044
**Water-COF$_{\text{TAPT-DHTA}}$**		-5.547	-2.826	2.721	
**COF$_{\text{TAPPy-DHTA}}$**	eclipsed AA	-5.504	-2.394	3.110	-0.132
**Water-COF$_{\text{TAPPy-DHTA}}$**		-5.416	-2.438	2.978	

Table S3 Raman shift assignment of $COF_{X\text{-}DHTA}$ films

**Samples**	**No. of Peaks**	**Raman Shift Dry (cm^{-1})**	**Raman Shift Wet (cm^{-1})**
**$COF_{TAPB\text{-}DHTA}$ Film**	v(C–N) or (C–C) Aromatic	1143.98	——
	v(C–N) or (C–C) Aromatic	1178.54	1167.04
	δC–H wag	1291.19	——
	v(C–N)	1392.07	1344.93
	υ(CC) aromatic ring chain vibrations	1545.97	1562.85
	V(C=N) Strong	1597.68	1590.49
	V(C–O)	——	——
**$COF_{TAPT\text{-}DHTA}$ Film**	v(C–N) or (C−C) Aromatic	1132.42	1128.56
	v(C–N) or (C–C) Aromatic	1187.47	1187.47
	δC–H wag	1226.87	1225.61
	v(C–N)	1327.48	1329.98
	υ(CC) aromatic ring chain vibrations	1548.39	1518.15
	V(C=N) Strong	1598.87	1598.87
	V(C–O)	1665.58	1665.58
**$COF_{TAPPy\text{-}DHTA}$ Film**	v(C–N) or (C–C) Aromatic	1131.13	1131.13
	v(C–N) or (C–C) Aromatic	1182.37	1182.37
	δC–H wag	1218.00	1218.00
	v(C–N)	1328.73	1328.73
	υ(CC) aromatic ring chain vibrations	——	1548.97
	V(C=N) Strong	1598.87	1598.87
	V(C–O)	1666.77	1665.58

Supplementary References

[S1] D. Zhu, R. Verduzco, Ultralow surface tension solvents enable facile COF activation with reduced pore collapse. ACS Appl. Mater. Interfaces **12**, 33121–33127 (2020). https://pubs.acs.org/doi/10.1021/acsami.0c09173

[S2] X. Yan, Y.Song, J. Liu, N. Zhou, C. Zhang et. al, Two-dimensional porphyrin-based covalent organic framework: A novel platform for sensitive epidermal growth factor receptor and living cancer cell detection. Biosens. Bioelectron. **126**, 734−742 (2019). https://doi.org/10.1016/j.bios.2018.11.047

[S3] B. P. Vinayan, R. Nagar, N. Rajalakshmi, S. Ramaprabhu, Novel platinum–cobalt alloy nanoparticles dispersed on nitrogen-doped graphene as a cathode electrocatalyst for PEMFC applications. Adv. Funct. Mater. **22**, 3519–3526 (2012). https://doi.org/10.1002/adfm.20110254

[S4] X. Hu, Z. Zhan, J. Zhang, I. Hussain, B. Tan, Immobilized covalent triazine frameworks films as effective photocatalysts for hydrogen evolution reaction. Nat. Commun. **12**, 6596 (2021). https://doi.org/10.1038/s41467-021-26817-4

[S5] P. Jagdale, D. Ziegler, M. Rovere, J. M. Tulliani, A. Tagliaferro, Waste coffee ground biochar: A material for humidity sensors. Sens. **19**, 801 (2019). https://doi.org/10.3390/s19040801

[S6] S. Lee, B. Choi, J. Kim, B. Woo, G. Kim, Reducing individual difference and temperature dependency of QCM humidity sensors based on graphene oxides through normalization of frequency shifts. Sens. Actuators Chem. B **313**, 128043 (2020). https://doi.org/10.1016/j.snb.2020.128043

[S7] L. Lan, X. Le, H. Dong, J. Xie, Y. Ying et al., One-step and large-scale fabrication of flexible and wearable humidity sensor based on laser-induced graphene for real-time tracking of plant transpiration at bio-interface. Biosens. Bioelectron. **165**, 112360 (2020). https://doi.org/10.1016/j.bios.2020.112360

[S8] P. Zhu, H. Ou, Y. Kuang, L. Hao, J. Diao et al., Cellulose nanofiber/carbon nanotube dual network-enabled humidity sensor with high sensitivity and durability. ACS Appl. Mater. Interfaces **12**(29), 33229–33238 (2020). https://doi.org/10.1021/acsami.0c07995

[S9] T. Li, L. Li, H. Sun, Y. Xu, X. Wang et al., Porous ionic membrane based flexible humidity sensor and its multifunctional applications. Adv. Sci. **4**(5), 1600404 (2017). https://doi.org/10.1002/advs.201600404

[S10] Z. Wu, X. Yang, J. Wu, Conductive hydrogel- and organohydrogel-based stretchable sensors. ACS Appl. Mater. Interfaces **13**(2), 2128–2144 (2021). https://doi.org/10.1021/acsami.0c21841

[S11] S. Rauf, T. Vijjapu, A. Andrés, I. Gascón, O. Roubeau et al., Highly selective metal-organic framework textile humidity sensor. ACS Appl. Mater. Interfaces **12**(26), 29999–30006 (2020). https://doi.org/10.1021/acsami.0c07532

[S12] A. Garg, M. Almáši, J. Bednarčík, R. Sharma, S. Rao et al., Gd(III) metal-organic framework as an effective humidity sensor and its hydrogen adsorption properties. Chemosphere **305**, 135467 (2022). https://doi.org/10.1016/j.chemosphere.2022.135467

[S13] J. Zhao, N. Li, H. Yu, Z. Wei, M. Liao et al., Highly sensitive MoS_2 humidity sensors array for noncontact sensation. Adv. Mater. **29**(34), 1702076 (2017). https://doi.org/10.1002/adma.201702076

[S14] H. Guo, C. Lan, Z. Zhou, P. Sun, D. Wei et al., Transparent, flexible, and stretchable WS_2 based humidity sensors for electronic skin. Nanoscale **9**(19), 6246–6253 (2017). https://doi.org/10.1039/c7nr01016h

[S15] U. Haq, R. Ullah, S. Mazhar, R. Khattak, A. Qarni et al., Synthesis and characterization of 2D Mxene: Device fabrication for humidity sensing. J. Sci.: Adv. Mater. Devices **7**(1), 100390 (2022). https://doi.org/10.1016/j.jsamd.2021.08.003

[S16] S. Muckley, M. Naguib, N. Ivanov, Multi-Mmodal, ultrasensitive, wide-range humidity sensing with Ti_3C_2 film. Nanoscale **10**(46), 21689–21695 (2018). https://doi.org/10.1039/c8nr05170d

[S17] Y. Zhang, W. Zhang, Q. Li, C. Chen, Z. Zhang, Design and fabrication of a novel humidity sensor based on ionic covalent organic framework. Sens. Actuators Chem. B **324**, 128733 (2020). https://doi.org/10.1016/j.snb.2020.128733

[S18] H. Singh, K. Omer, N, Jena, I. Bala, N. Sharma et al., A porous, crystalline truxene-based covalent organic framework and its application in humidity sensing. J. Mater. Chem. A **5**(41), 21820–21827 (2017). https://doi.org/10.1039/C7TA05043G

[S19] L. Ascherl, E. Evans, M. Hennemann, D. Nuzzo, G. Hufnagel et al., Solvatochromic covalent organic frameworks. Nat. Commun. **9**(1), 3802 (2018). https://doi.org/10.1038/s41467-018-06161-w

[S20] W. Huang, Y. Jiang, X.J. Li, X. Li, J.Y. Wang et al., Solvothermal synthesis of microporous, crystalline covalent organic framework nanofibers and their colorimetric nanohybrid structures. ACS Appl. Mater. Interfaces **5**(18), 8845–8849. (2013). https://doi.org/10.1021/am402649g

[S21] J. Feng, X. Kang, Q. Zuo, C. Yuan, W. Wang et al., Fabrication and evaluation of a graphene oxide-based capacitive humidity sensor. Sensors **16**(3), 314 (2016). https://doi.org/10.3390/s16030314

[S22] J. Boudaden, M. Steinmaßl, H. Endres, A. Drost, I. Eisele et al., Polyimide-based capacitive humidity sensor. Sensors **18**(5), 1516 (2018). https://doi.org/10.3390/s18051516

[S23] P. Shuk, M. Greenblat, Solid electrolyte film humidity sensor. Solid State Ionics **113**(115), 229–233 (1998). https://doi.org/10.1016/S0167-2738(98)00376-2
